# Regulation of mRNA export through API5 and nuclear FGF2 interaction

**DOI:** 10.1093/nar/gkaa335

**Published:** 2020-05-08

**Authors:** Seoung Min Bong, Seung-Hyun Bae, Bomin Song, HyeRan Gwak, Seung-Won Yang, Sunshin Kim, Seungyoon Nam, Krishnaraj Rajalingam, Se Jin Oh, Tae Woo Kim, SangYoun Park, Hyonchol Jang, Byung Il Lee

**Affiliations:** 1 Research Institute, National Cancer Center, Goyang-si, Gyeonggi 10408, Republic of Korea; 2 Department of Cancer Biomedical Science, National Cancer Center Graduate School of Cancer Science and Policy, Goyang-si, Gyeonggi 10408, Republic of Korea; 3 Department of Life Sciences, College of BioNano Technology and Department of Genome Medicine and Science, Graduate School of Medicine, Gachon University, Incheon 21565, Republic of Korea; 4 Cell Biology Unit, University Medical Center Mainz, JGU, Mainz, Germany; 5 Department of Biomedical Sciences, Graduate School of Medicine, Korea University, Seoul 02841, Republic of Korea; 6 School of Systems Biomedical Science, Soongsil University, Seoul 06978, Republic of Korea

## Abstract

API5 (APoptosis Inhibitor 5) and nuclear FGF2 (Fibroblast Growth Factor 2) are upregulated in various human cancers and are correlated with poor prognosis. Although their physical interaction has been identified, the function related to the resulting complex is unknown. Here, we determined the crystal structure of the API5–FGF2 complex and identified critical residues driving the protein interaction. These findings provided a structural basis for the nuclear localization of the FGF2 isoform lacking a canonical nuclear localization signal and identified a cryptic nuclear localization sequence in FGF2. The interaction between API5 and FGF2 was important for mRNA nuclear export through both the TREX and eIF4E/LRPPRC mRNA export complexes, thus regulating the export of bulk mRNA and specific mRNAs containing eIF4E sensitivity elements, such as c-MYC and cyclin D1. These data show the newly identified molecular function of API5 and nuclear FGF2, and provide a clue to understanding the dynamic regulation of mRNA export.

## INTRODUCTION

Apoptosis inhibitor 5 (API5, also called AAC-11 or FIF) is a nuclear protein that inhibits apoptosis in human cells. This protein was originally identified in surviving cells after serum deprivation and was later found to be upregulated in various cancers (1–4). Recent studies have suggested that API5 is important for cell cycle progression (5), immune escape (6), metastasis (7), and the stem-cell-like properties of cancer cells (8) and that it promotes drug resistance in cancers (9,10). Molecular mechanistic studies have shown that API5 prevents cell death by negatively regulating E2F1 transcription factor-induced apoptosis (11), protecting acinus from caspase 3 cleavage (10), inhibiting caspase 2 (12), or degrading the pro-apoptotic protein BIM through the FGF2–FGFR1–PKCδ–Erk signaling pathway (6). The crystal structure of API5 suggests that it functions as a protein-protein interaction mediator with HEAT (at the N-terminal half) and ARM-like (at the C-terminal half) repeat protein binding modules (13). Several interaction partners have been identified, including fibroblast growth factor 2 (FGF2) (14), acinus (10), influenza A virus nucleoprotein (15), estrogen receptor α (ERα) (16) and caspase 2 (12). However, the functions of these interactions are poorly understood, in part due to the lack of structural information.

Here, we focused on the API5–FGF2 interaction (14). FGF2 is a well-known mitogenic growth factor (17). Among the five isoforms of human FGF2, a low-molecular-weight (LMW) isoform lacking the N-terminal extensions is usually secreted to function in autocrine or paracrine FGF2 signaling by association with heparan sulfate proteoglycans (HSPGs) and FGF receptors (FGFRs) (17). However, a large amount of LMW FGF2 can also localize in the nucleus via a noncanonical cryptic nuclear localization signal (NLS) (18). High-molecular-weight (HMW) FGF2 isoforms that possess N-terminal NLS sequences are localized to the nucleus to perform various FGFR-independent functions (19). Originally, HMW FGF2 isoforms were identified as interaction partners of API5 (14). Subsequently, however, the LMW FGF2 isoform was also found to interact with API5 *in vitro* (13). Because API5 is a nuclear protein, the physical interaction between API5 and FGF2 seems to be determined by cellular localization rather than the intrinsic properties of the FGF2 isoforms *in vivo*. While FGF2 signaling through secreted FGF2 is well understood, the cellular functions and molecular mechanisms of nuclear FGF2 have not been well studied, even though upregulation of nuclear FGF2 is highly correlated with poor prognosis and metastasis in cancers (20,21).

We reasoned that structural and functional studies of the API5–FGF2 complex would reveal insights into the contributions of these two proteins to cancer. We therefore determined the crystal structure of the API5–FGF2 complex. The structure provided a detailed picture of the protein-protein interactions, and we identified the specific residues involved. By integrating structural, proteomic, and functional studies, we unexpectedly found that API5 and FGF2 function in mRNA export in mammalian cells by directly interacting with UAP56 (ATP-dependent RNA helicase), an essential component of various mRNA export machineries, and thereby regulate the expression of several specific oncogenes. Our results provide new insight into the molecular function and mechanism of API5 and nuclear FGF2 as novel components of the mRNA export machineries and suggest a new therapeutic opportunity for the regulation of oncogenic gene expression in cancers.

## MATERIALS AND METHODS

### Expression constructs

All expression constructs for this study are summarized in Supplementary Table S1. To prepare API5 recombinant protein using an *Escherichia coli* expression system, the PCR-amplified human *API5* gene (covering residues 1–504, isoform 2) was inserted into the expression vector pET-28b(+) (Novagen, USA). For FGF2 overexpression in *E. coli*, the codon-optimized *FGF2* gene encoding LMW FGF2 (residues 135–288; C211S/C229S mutant which corresponds to the C69S/C87S mutant in previously reported FGF2 structures) was chemically synthesized (COSMO Genetech, Korea) and cloned into a modified pET-28b(+) vector. The GST-API5 and GST-UAP56 constructs were cloned into the pGEX-4T-3 (GE Healthcare, USA) vector. For protein expression of the wild-type and mutant *API5* genes in mammalian cells, PCR-amplified *API5* WT and mutant genes were inserted into the pCAG-F-BS (pCAG-FLAG-IRES-blasticidin) vector. The LMW *FGF2* WT or mutant genes were cloned into the pCAG-HA-puro (pCAG-HA-IRES-puromycin) vector. For the lentiviral short hairpin RNA (shRNA)-mediated conditional knockdown of *API5* or *FGF2*, the targeting sequences were inserted into the Tet-pLKO-puro vector (a gift from Dmitri Wiederschain, Addgene plasmid # 21915). For the lentiviral CRISPR/Cas9-mediated knockout of *API5*, guide RNA sequences targeting *API5* were inserted into the lentiCRISPR v2 vector (a gift from Feng Zhang, Addgene plasmid # 52961). Lentiviral constructs for the expression of API5-derived peptide were constructed by cloning synthesized DNA sequences into the pUltra vector (a gift from Malcolm Moore, Addgene plasmid # 24129). The lentivirus-mediated peptide expression was monitored by the GFP fluorescence signal. All information on shRNA and guide RNA sequences for knockdown of each gene are also summarized in Supplementary Table S1.

### Protein expression, purification, crystallization and crystal structure determination

Protein expression, purification, and crystallization experiments were performed as described elsewhere (22). Briefly, each protein was overexpressed in the *E. coli* Rosetta2(DE3) strain at 37°C for API5 or 18°C for FGF2 (Novagen, USA). Each protein was purified using a Ni-NTA resin (Qiagen, Germany) and a HiLoad 16/600 Superdex 200 or 75 prep grade column (GE Healthcare, USA). Purified API5 and FGF2 were mixed together and incubated at 4°C overnight to form the API5–FGF2 complex. The molar ratio of API5 to FGF2 was 1:3. Crystals were obtained in a reservoir solution of 100 mM Na-HEPES (pH 7.5), 100 mM KCl, and 10% (v/v) polyethylene glycol (PEG) 6000. X-ray diffraction data were collected using an ADSC Q270 detector at beamline 7A of Pohang Light Source (Pohang, Korea). The initial crystal structure of the API5–FGF2 complex was determined by a molecular replacement method using the MOLREP program in the CCP4 program package with PDB code 3U0R (for API5) (13) and PDB code 1BAS (for FGF2) (23). Manual model building was completed using the program COOT (24). The program PHENIX (25) was used for model refinement. The detailed data collection and refinement statistics are summarized in Table 1.

**Table 1.** Data collection and refinement statistics

**Data collection**	
X-ray source	PLS-7A
X-ray wavelength (Å)	1.000
Space group	*P*2_1_2_1_2_1_
Unit cell parameters (Å)	*a* = 46.862, *b* = 76.523, *c* = 208.158
Resolution range (Å)	50.0−2.60 (2.64−2.60)^a^
Total/unique reflections	101,039/22,727
Completeness (%)	94.5 (81.9)
*I*/σ*I*	15.7 (2.1)
*R*_merge_ (%)^b^	10.5 (37.5)
Redundancy	4.4 (2.2)
**Model refinement**	
*R*_work_/*R*_free_^c^	0.222/0.260
Number of nonhydrogen atoms	
Protein/water	4,455/35
Average *B* factor (Å^2^)	
Protein/water	53.4/39.4
R.m.s. deviations	
Bond lengths (Å)/angles (°)	0.001/0.400
Ramachandran plot (%)	
Favored/outliers	96.36/0.00
Rotamer outliers (%)	0.20
Overall MolProbity score	1.45
PDB code	6L4O

^a^Value in parentheses are for the highest-resolution shell.

^b^ *R*_merge_ = Σ_*h*_ Σ_*i*_ | *I*(*h*)_*i*_ – < *I*(*h*) > | / Σ_*h*_ Σ_*i*_*I*(*h*)_*i*_, where *I*(*h*) is the intensity for reflection *h*, Σ_*h*_ is the sum for all reflections, and Σ_*i*_ is the sum for *i* measurements of reflection *h*.

^c^ *R* = Σ | |*F*_obs_| – |*F*_calc_| | / Σ |*F*_obs_|, where *R*_free_ is calculated for a randomly chosen 5% of reflections, which were not used for structure refinement, and *R*_work_ is calculated for the remaining reflections.

### Bulk mRNA export assay by RNA-FISH

Cells were cultured in MEM (HyClone, USA) supplemented with 10% FBS and 1% penicillin–streptomycin (Gibco, USA) and grown in the presence or absence of doxycycline for 4 days. Actinomycin D (10 μM final concentration, Gibco, USA) was added 2 h before cell fixation to reduce nascent RNA signals as described in previous reports (26,27). For fluorescence *in situ* hybridization (FISH) experiments, a Stellaris^®^ RNA-FISH Kit (LGC Bioresearch Technologies, USA) was used, and all experiments were performed according to the manufacturer's instructions. Cy3-labeled oligo(dT)_50_ was used as the probe (Gene Link, USA) for the detection of bulk mRNA. Briefly, after fixation, cells were prewashed with 1× wash buffer A from the RNA-FISH kit, supplemented with 10% formamide. For hybridization, 125 nM of FISH probes dissolved in hybridization buffer containing 10% formamide were used. Samples were hybridized in the dark at 37°C for 4 h. Following hybridization, samples were washed with 1 ml of wash buffer A supplemented with 10% formamide for 30 min at 37°C. Next, 1 ml of 5 ng/ml DAPI dissolved in 1× wash buffer A supplemented with 10% formamide was added to counterstain nuclei for 30 min at 37°C, and each well was washed with 1 ml of wash buffer B from the RNA-FISH kit for 5 min at room temperature. Samples were mounted with Vectashield Mounting Medium (Invitrogen, USA) on a microscope slide, and poly(A)^+^ RNA signals were detected. Images were obtained under confocal II LSM780 (Carl Zeiss, Germany) and were processed and quantified using the ZEN 2012 program (Carl Zeiss, Germany).

### Analysis of nuclear/cytosolic RNA levels by RT-qPCR

To determine the ratio of nuclear to cytosolic mRNA for specific genes, subcellular RNA fractionation was carried out using an RNA Subcellular Isolation Kit (Active Motif, USA) according to the manufacturer's instructions. The reverse transcription PCR and the real-time quantitative PCR (RT-qPCR) were performed according to a modified version of the method described previously (28). Briefly, cDNA was synthesized using AMV Reverse Transcriptase (Takara Bio, Japan), and RT-qPCR was performed with a FastStart Essential DNA Green Master Kit (Roche Diagnostics, USA) using a Real-time PCR LightCycler 96 (Roche Diagnostics, Switzerland). The MIQE (29) checklist is provided in Supplementary Table S2.

### Other biochemical and cellular experiments

Other biochemical and cellular experiments were performed as described in Supplementary Materials and Methods. The antibodies used in this study are summarized in Supplementary Table S3. Full immunoblots are shown in Supplementary Figure S1. Cell lines were authenticated by short tandem repeat (STR) profiling by the GenePrint® 10 System (Promega, USA) and regularly checked for *Mycoplasma* by the Universal Mycoplasma Detection Kit (ATCC^®^ 30-1012KTM) at the Genomics Core (National Cancer Center, Korea).

### Statistics

The data are presented as the means ± standard deviation, and *P* values calculated using a Student's t-test calculator (http://graphpad.com/quickcalcs/). The data are representative of at least three separate experiments.

## RESULTS

### The crystal structure of the API5–FGF2 complex reveals residues critical for the interaction

**Figure 1.**
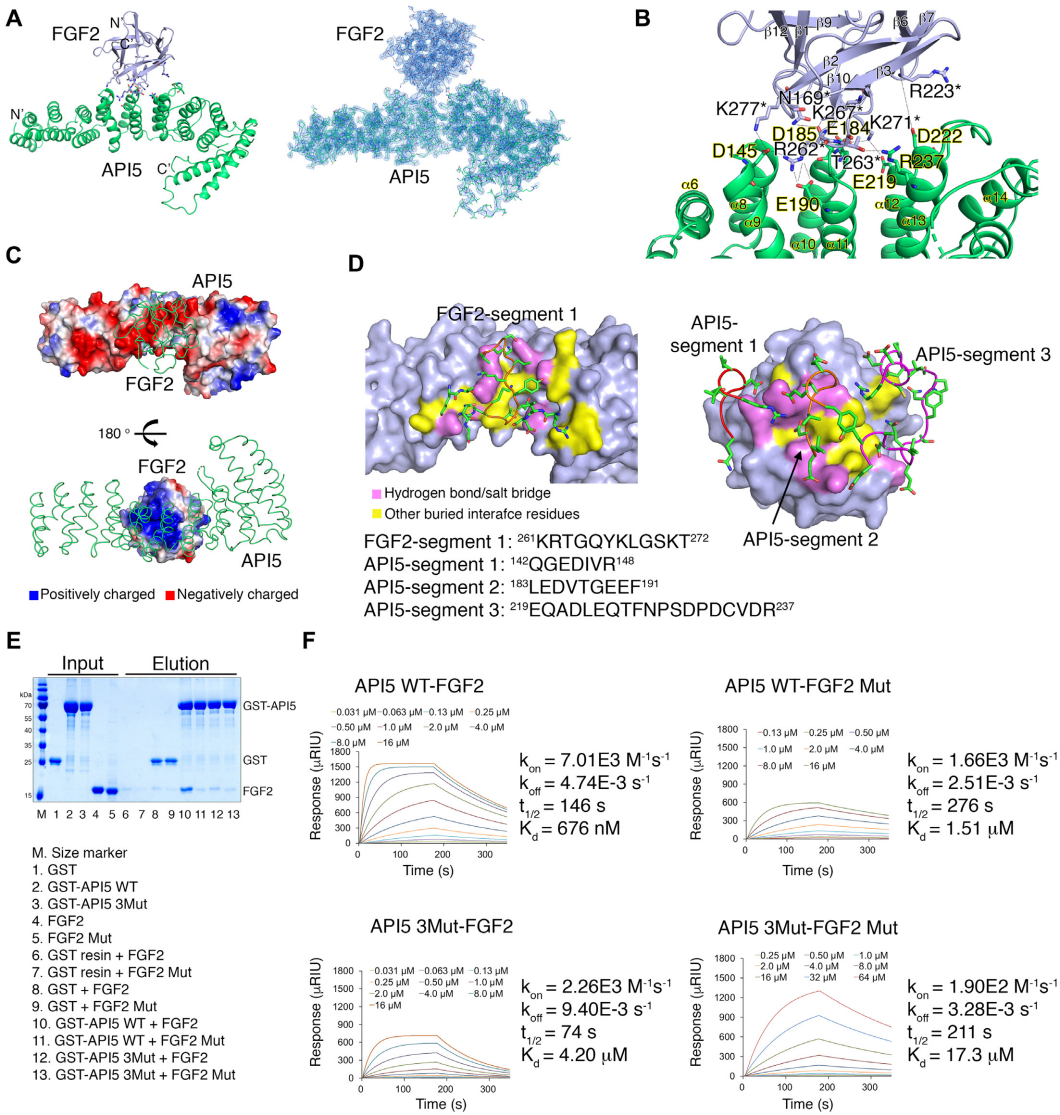
Crystal structure of the API5–FGF2 complex and validation of the API5–FGF2 interaction. (**A**) Left, overall structure of the API5–FGF2 complex. The N- and C-termini of each protein are indicated by N’ and C’, respectively. Structure of API5 are colored in green and the FGF2 structure are colored in light blue. Right, electron density map of the API5–FGF2 complex. (**B**) Detailed view of the protein-protein interaction interface. Residues participating in the hydrogen bonds and salt bridges in the protein interaction are shown. Residues of FGF2 are marked with asterisks. (**C**) Electrostatic surface potential of API5 and FGF2. Positively charged residues are colored in blue, negatively charged residues are colored in red, and neutral residues are represented in white. (**D**) Segments of FGF2 and API5 participating in the interaction. One major basic segment from FGF2 (FGF2-segment 1) and three major acidic segments from API5 (API5-segment1, API5-segment 2, and API5-segment3) participate in the protein-protein interaction. In the API5–FGF2 interaction interface, the residues involved in hydrogen bonding or salt bridges are colored in pink and other buried interface residues are colored in yellow. (**E**) GST pulldown with purified recombinant GST-API5 and His-FGF2. Protein bands were visualized by the Coomassie blue staining method. (**F**) SPR experiments with purified recombinant His-API5 and His-FGF2. The *k*_on_ is the association rate constant (M^−1^ s^−1^), *k*_off_ is the dissociation rate constant (s^−1^), *t*_1/2_ is the half-life of complex (s), and *K*_d_ is the equilibrium dissociation constant (M).

To investigate the function of the API5–FGF2 complex and its potential role in cancer, we determined the crystal structure of the API5–LMW FGF2 complex at a resolution of 2.6 Å (Figure 1A; Table 1). The obtained structure revealed that FGF2 binds to the central region of the API5 helical repeat and provided a detailed picture of the protein-protein interaction interface (Figure 1B). There were few structural changes from the previously determined API5- or FGF2-only structures when the two proteins interacted with each other (Supplementary Figure S2), suggesting that the interface structures are preformed and structurally rigid: i.e. formed by conformational selection mechanism rather than interacting through induced fit. The interface surface area between FGF2 and API5 was relatively small – approximately 555 Å^2^ (2.5% and 8.4% of the total surface areas of API5 and FGF2, respectively) – compared to approximately 1300 Å^2^ of the interface area between FGF2 and FGFR1 (PDB entry 1FQ9) (30) (Supplementary Tables S4 and S5). The major interaction force between the interface is the multiple electrostatic interactions when the surface charge distribution of the interface areas and salt-dependent decrease of the API5–FGF2 interaction (Figure 1C; Supplementary Figure S3) are considered. Twenty residues in API5 and 14 residues in FGF2 directly participate in protein-protein interactions. Among these, seven highly conserved mainly negatively charged residues in API5 (Asp145 in the α8−α9 loop, Glu184/Asp185 in the α10−α11 loop, Glu190 in α11, Glu219 in α12, Asp222 in the α12−α13 loop and Arg237 in α13) form hydrogen bonds or salt bridges with FGF2 (Figure 1B; Supplementary Table S4). This region in API5 corresponds to the ‘convex’ side of the structure, which is also the central region of the API5 helical repeat that connects the HEAT (α1−α11) and ARM-like (α12−α19) helical repeats (13). Seven positively charged residues on the surface of FGF2 (Asn169 in the β1−β2 loop, Arg223 in β7, Arg262/Thr263 in the β10−β11 loop; Lys 267 in β11; and Lys271 and Lys277 in the β11−β12 loop) form hydrogen bonds or salt bridges with API5 (Figure 1B; Supplementary Table S4). Seven other residues of FGF2 (Gly170, Arg181, Lys261, Gln265, Tyr266, Leu268 and Ala278) and 13 residues of API5 (Gly143, Glu144, Arg148, Leu183, Val186, Thr187, Gly188, Gln220, Glu224, Gln225, Asn228, Ser230 and Asp231) form an additional interface for the API5–FGF2 interaction. Taken together, the interacting residues can be divided into segments of highly conserved basic (FGF2) or acidic (API5) regions (FGF2-segment 1, ^261^KRTGQYKLGSKT^272^; API5-segment 1, ^142^QGEDIVR^148^; API5-segment 2, ^183^LEDVTGEEF^191^; and API5-segment 3, ^219^EQADLEQTFNPSDPDCVDR^237^) (Figure 1D; Supplementary Figure S4).

We further tested the API5–FGF2 interaction by GST pulldown and surface plasmon resonance (SPR) with recombinant API5 and FGF2 (Figure 1E and F), revealing direct physical interaction between wild-type (WT) of API5 and FGF2 (C211S/C229S which structurally similar to WT, note that C211S/C229S mutation was only for the stable expression of recombinant FGF2 in *E. coli* and not related in API5 interaction). In both the GST pulldown and SPR studies, the strength of the WT API5–FGF2 interaction was considerably decreased when the residues involved in the protein–protein interaction in API5-segment 2 and FGF2-segment 1 were mutated. While the *K*_d_ value determined by SPR for WT API5–FGF2 interaction was 676(±5) nM, the *K*_d_ values for API5 WT–FGF2 Mut (FGF2-segment 1 mutation, C211S/C229S/R262A/T263A/K271A), API5 3Mut (API5-segment 2 mutation, E184A/D185A/E190A)–FGF2, and API5 3Mut–FGF2 Mut were 1.51(±0.01), 4.2(±0.1), and 17.3(±0.4) μM, respectively. The decreased affinities in case of the interface mutations were mainly by reduced association rates (*k*_on_). The detailed binding affinities and kinetic parameters are summarized in Supplementary Table S6.

### API5-interacting region in FGF2 overlaps with the heparin-binding region

**Figure 2.**
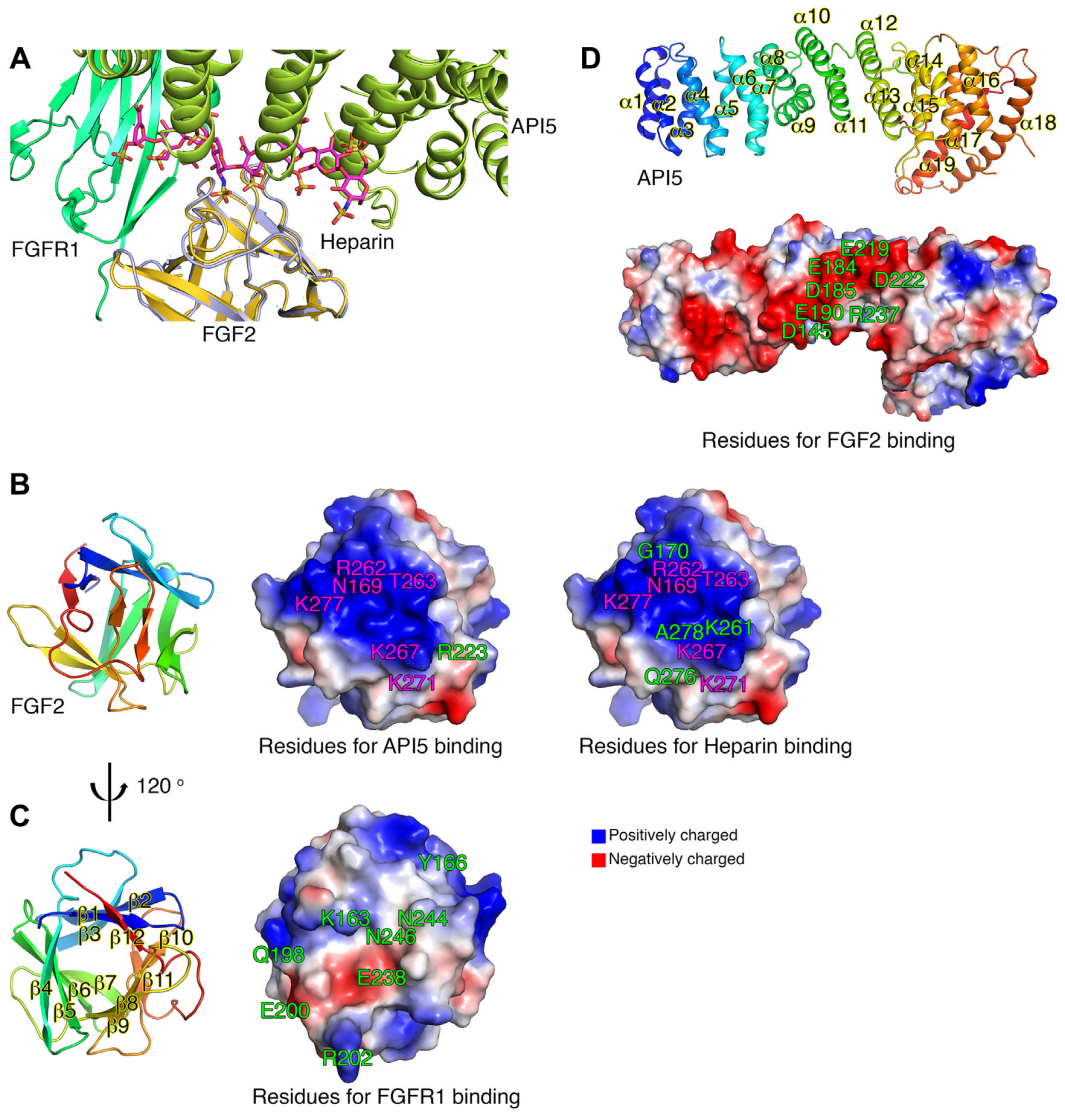
Structural analysis of the API5–FGF2 complex. (**A**) Detailed view of the structural superposition of API5–FGF2 on FGF2–FGFR1–heparin (PDB entry 1FQ9) with the FGF2 structure as the central figure. The heparin molecule in the FGF2–FGFR1–heparin complex is colored in magenta and API5 from the API5–FGF2 complex is drawn in limon. FGFR1 is drawn in green. FGF2 in the FGF2–FGFR1–heparin complex is drawn in yellow orange and FGF2 in the API5–FGF2 complex is drawn in light blue. (**B**) Residues of FGF2 forming hydrogen bonds or salt bridges with API5 or heparin. Residues of FGF2 involved in both API5 and heparin binding are colored in magenta, and residues of FGF2 participating either API5 or heparin interaction are colored in green. (**C**) Residues of FGF2 forming hydrogen bonds or salt bridges with FGFR1 are colored in green. (**D**) Residues of API5 forming hydrogen bonds or salt bridges with FGF2. Overall structures of API5 and FGF2 are shown to present the orientation of each molecule. All interface residues of FGF2 with API5, heparin and FGFR1 are summarized in Supplementary Tables S4 and S5.

On the cell surface, secreted FGF2 usually performs its biological functions by activating FGFRs. The dimerization of FGF2-bound FGFR is a prerequisite for the activation of FGFRs, and HSPG plays a critical role in FGF2 signaling by forming a ternary complex with FGF2 and FGFR (17). A structural study of the FGF2–FGFR1–heparin complex suggested the ‘two-end model’ for its dimerization and activation mechanism (30). Although the cellular localization sites of API5–FGF2 (nucleus) and FGF2–FGFR1–heparin (cell surface) are different, we compared the structure of the API5–FGF2 complex with the structure of the FGF2–FGFR1–heparin complex to obtain clues as to the functions of API5 and nuclear FGF2. When the API5–FGF2 complex structure was superimposed to that of the FGF2–FGFR1–heparin ternary complex with FGF2 as the central figure, the API5 structure was positioned at the heparin region of the ternary complex (Figure 2A; Supplementary Figure S5). Moreover, when the FGFR1–FGF2 structure was shown in the dimeric state, there were many steric clashes with the API5–FGF2 structure, which would be hard to overcome even if a large conformational change occurred in the helical repeat structure of API5.

The API5-interacting region of FGF2 largely overlaps with the heparin-binding region of FGF2, and the FGFR1-binding region of FGF2 is located on the opposite side of FGF2 (Figure 2B and C; Supplementary Tables S4–5). The API5 residues involved in the FGF2 interaction are mostly negatively charged, similar to the surface properties of heparin (Figures 1C; 2D). Compared to the API5–FGF2 complex, additional hydrogen bonds or salt bridges were found in FGF2–heparin complex structures (Figure 2B; Supplementary Table S4). The residues of FGF2 that interact with heparin through hydrogen bonding or salt bridges are Asn169, Gly170 (in the β1−β2 loop), Lys261, Arg262, Thr263 (in the β10−β11 loop), Lys267 (in β11), and Lys271, Gln276, Lys277 and Ala278 (in the β11−β12 loop). The binding affinity of FGF2–heparin was stronger (the *K*_d_ = 4.4 (±0.5) nM, *k*_on_ = 5.45(±0.06) × 10^5^ M^−1^ s^−1^ and *k*_off_ = 2.38(±0.02) × 10^−3^ s^−1^) than the binding affinity of API5–FGF2 (676(±5) nM). Notably, the association rate was 100-fold higher in FGF2–heparin complex formation when compared to that of FGF2 and API5 interaction (Supplementary Figure S6).

### The API5−FGF2 interaction is important for the nuclear localization of LMW FGF2

**Figure 3.**
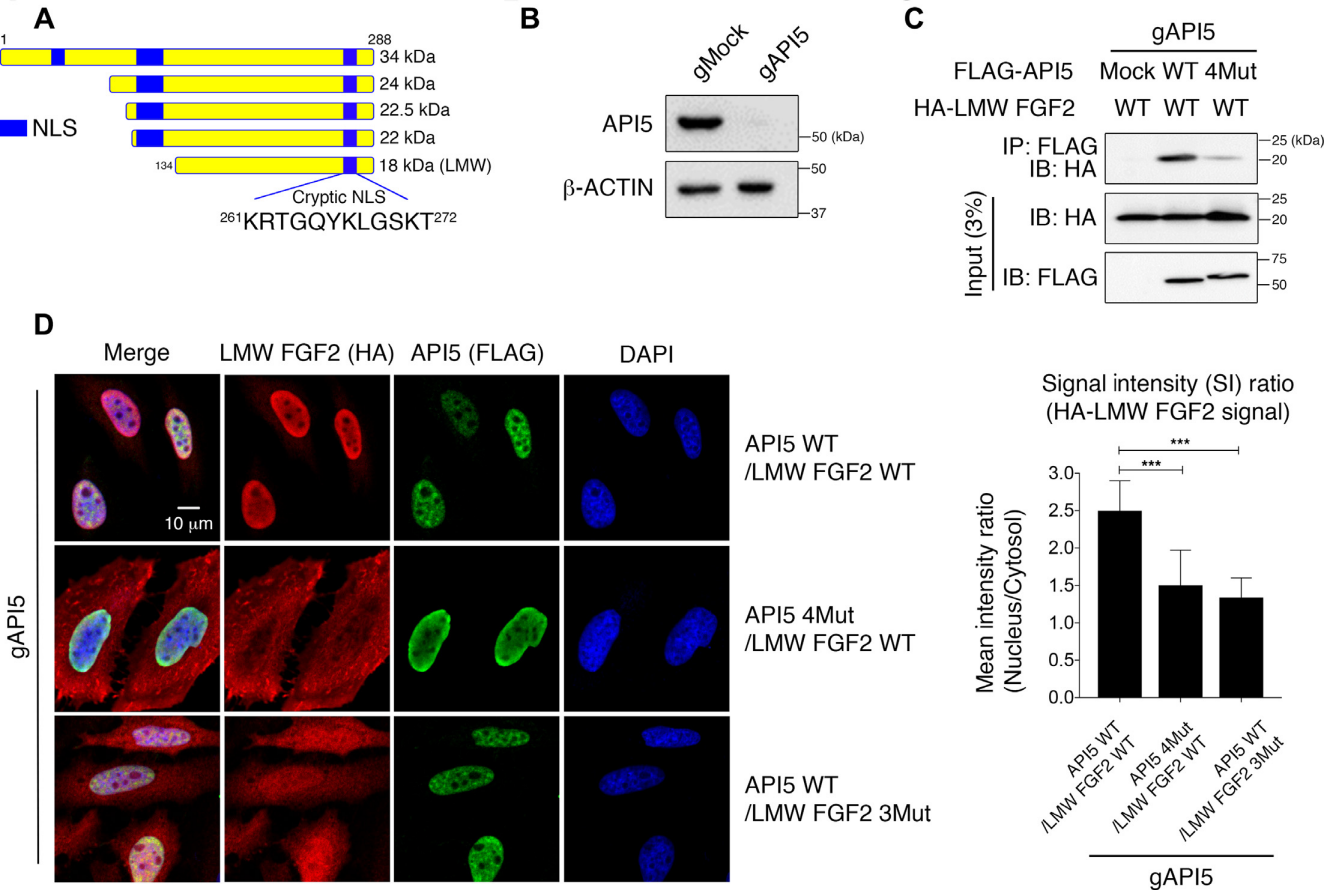
Cellular localization of LMW FGF2 by API5. (**A**) Schematic representation of five human FGF2 isoforms and the position of the cryptic NLS region. The cryptic NLS corresponds to the FGF2-segment 1. (**B**) Western blot validation of HeLa cells after *API5* knockout (gAPI5) by the CRISPR/Cas9 system. (**C**) Monitoring of intracellular interactions of API5 and LMW FGF2 by co-immunoprecipitation assay. Transiently co-expressed FLAG-API5 (WT or 4Mut) and HA-tagged LMW FGF2 WT in *API5* knockout (gAPI5) HeLa cells were used. (**D**) Cellular localization of API5 and LMW FGF2 observed by confocal microscopy and quantified mean signal intensity (SI) ratios of nucleus/cytosol (*n* = 46 for each sample). The error bars represent the mean ± SD, ***P < 0.001 (Student's *t*-test).

The primary amino acid sequence of LMW FGF2 shows that it does not have a canonical NLS sequence. Interestingly, two basic residues of FGF2 that participate in API5 or heparin binding, Lys261 and Arg262 of FGF2-segment 1, are part of the previously identified a noncanonical cryptic NLS in LMW FGF2 (18), which suggested that the mechanism of nuclear localization of LMW FGF2 may be through the API5−FGF2 interaction (Figure 3A). To prove this, we monitored the cellular localization of LMW FGF2 with regards to the API5−FGF2 interaction. To exclude the influence of the endogenous API5 WT, we generated API5 knockout cells using the CRISPR/Cas9 system (Figure 3B). Using these cells, both FLAG-tagged API5 and HA-tagged LMW FGF2 were co-expressed, and a co-immunoprecipitation assay using an anti-Flag antibody was performed. As expected, reduced interaction was shown when API5 was mutated (API5 4Mut; D145A/E184A/D185A/E190A) in the API5−FGF2 interaction interface (Figure 3C). To determine the consequences in the cellular localization of LMW FGF2 upon the physical interaction of two proteins, the HA-tagged LMW FGF2 WT or 3Mut (R262A/T263A/K271A; mutations in FGF2-segment 1) was transiently co-expressed with either API5 WT or 4Mut into the API5 knockout cells. Confocal microscopy experiments showed that while LMW FGF2 was distributed mainly in the nucleus when API5 WT and LMW FGF2 WT were expressed, the cytoplasmic amounts of LMW FGF2 increased when API5 4Mut or FGF2 3Mut were expressed instead (Figure 3D). These results show that the API5−FGF2 interaction is necessary for the nuclear localization of LMW FGF2, and that the cryptic NLS sequence is within our defined API5-interacting segment 1 of LMW FGF2 as identified in this study (Figure 3A).

### Interactome analysis with the API5–FGF2 complex suggests a functional link to mRNA export

**Figure 4.**
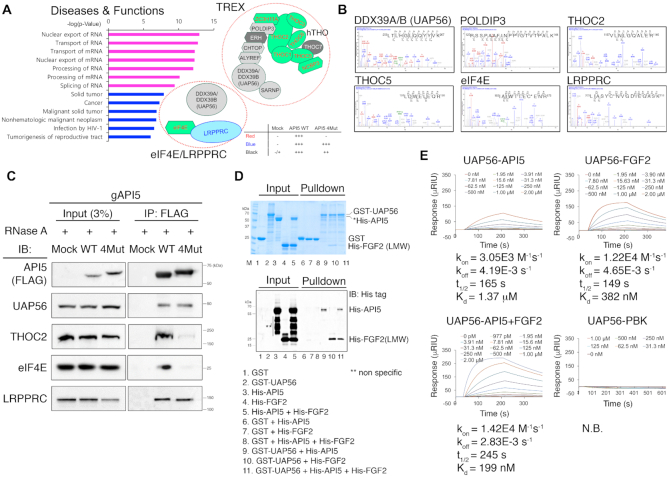
API5 is associated with the mRNA export machineries. (**A**) Left, disease–function annotations and pathways analyses of the API5 interaction partner list derived from immunoprecipitation/proteomics analysis using the IPA program. Top 7 pathways (*P* < 10^−9^) were shown in magenta. Right, API5 interaction partners that are members of the human TREX or eIF4E/LRPPRC mRNA export complex. API5–FGF2 interaction-dependent API5 interaction partners are shown in red text with green background (‘WT only’ in Table 2). API5–FGF2 interaction-independent API5 interaction partner is shown in blue text (‘All’ in Table 2). The proteins shown in black text are API5 interaction partners partially dependent on API5–FGF2 interaction (‘WT ↑’ in Table 2). ERH and THOC7 (in white text with grey background) which are known members of the TREX complex were not identified positive in our proteomics analysis. (**B**) Representative mass spectrometry data of identified API5-binding proteins related to the mRNA export machineries. (**C**) Validation of the interaction between API5 and mRNA export machinery components by immunoprecipitation. *API5* knockout (gAPI5) and reconstituted (WT or 4Mut) HeLa cells were used. RNase A was used during immunoprecipitation experiments to exclude any possible RNA-mediated interactions. (**D**) GST pulldown experiments with purified recombinant GST-UAP56, His-API5 and His-LMW FGF2 to monitor the direct interactions between UAP56 and API5, UAP56 and FGF2, and UAP56 and the API5–FGF2 complex. Protein bands were visualized both by Coomassie blue staining (up) and western blot analysis (down). Due to the contaminant protein bands of similar molecular weight to His-API5 (Marked in an asterisk, *), the His-API5 bands could not be distinguished clearly by Coomassie blue staining. Therefore, the His-API5 and His-FGF2 bands were validated by western blot with Anti-His tag antibody. The multiple bands marked in a double asterisk (**; lanes 3, 4 and 5) are likely to be non-specific bands. (**E**) Monitoring direct interactions between UAP56 and API5, UAP56 and FGF2, and UAP56 and the API5–FGF2 complex by SPR experiments. The concentrations of each analyte protein are indicated. The SPR experiment with UAP56 and PBK (PDZ-binding kinase) is the negative control (N.B.; no binding).

To gain further insights into the function of the API5–FGF2 complex, we explored interacting partners of API5 WT and API5 4Mut. To this end, we reconstituted FLAG-tagged API5 WT or 4Mut with similar expression levels in API5 knockout cells (Supplementary Figure S7). Immunoprecipitation and proteomics analysis (Supplementary Figure S7) provided large sets of API5 interaction partners. Among the 1408 proteins identified by proteomics analysis, 235 proteins were identified as API5-specific interaction partners (Supplementary Table S7). The FGF2 peptide was found only in the API5 WT sample, suggesting that the immunoprecipitation experiments and proteomics analysis were performed rigorously (Table 2). Pathway analysis using the API5-interacting proteins suggested a functional link to mRNA processing and export (Figure 4A; Supplementary Figure S8; Table 2; Supplementary Table S8). Several proteins in the mRNA export machineries, such as the TREX complex in the NXF1-dependent pathway and the eIF4E/LRPPRC complex in the CRM1-dependent pathway, were identified (31,32) (Figure 4A and B; Table 2). Nine known component proteins (THOC1, THOC2, THOC3, THOC5, THOC6, ZC3H11A, NCBP3, FYTTD1 (UIF) and eIF4E) in the human TREX and eIF4E/LRPPRC complexes were identified as API5−FGF2 interaction-dependent API5 binding proteins (Figure 4A; Table 2). Six additional TREX components (DDX39A, UAP56(DDX39B), POLDIP3, ALYREF, CHTOP, and SARNP) were identified as API5 interaction partners partially dependent on API5-FGF2 interaction and LRPPRC were identified as an API5−FGF2 interaction-independent API5 interaction partner (Figure 4A; Table 2).

**Table 2.** mRNA export machinery proteins identified by immunoprecipitation and mass spectrometry

Name	Aliases^a^	Uniprot entry	Total^b^	Unique^c^	Etc.^d^
FGF2	bFGF, HBGF-2	P09038	1	1	WT only
**NXF1 pathway**					
THOC1	HPR1, p84N5	Q96FV9	7	7	WT only
THOC2	THO2	Q8NI27	10	10	WT only
THOC3	THO3	Q96J01	1	1	WT only
THOC5	fSAP79, PP32.9	Q13769	3	3	WT only
THOC6	fSAP35, WDR58	Q86W42	4	4	WT only
ZC3H11A	KIAA0663, ZC3HDC11A	O75152	14	14	WT only
POLDIP3	SKAR, PDIP46	Q9BY77	17	1	WT ↑
NCBP3	C17orf85, ELG	Q53F19	8	8	WT only
FYTTD1	UIF	Q96QD9	8	8	WT only
DDX39A	DDX39	O00148	18	5	WT ↑
DDX39B	BAT1, UAP56	Q13838	21	8	WT ↑
SARNP	CIP29, HCC-1	P82979	7	7	WT ↑
ALYREF	THO4, Aly/REF	Q86V81	5	5	WT ↑
CHTOP	SRAG	Q9Y3Y2	5	5	WT ↑
**CRM pathway**					
eIF4E	EIF4EL1, EIF4F	P06730	2	2	WT only
LRPPRC	LRP130, GP130	P42704	7	7	All

^a^Referred to UniProt database (https://www.uniprot.org/).

^b^Number of total peptides.

^c^Number of unique peptides.

^d^WT only: API5−FGF2 interaction-dependent API5 interaction partners (Mock: Not found, API5 WT: High, API5 4Mut: Not found), WT ↑: API5 interaction partners partially dependent on API5-FGF2 interaction (Mock: Not found/Low, API5 WT: High, API5 4Mut: High, Area ratios for API5 WT/API5 4Mut were between 4 to 20), All: API5−FGF2 interaction-independent API5 interaction partner (Mock: Not found, API5 WT: High, API5 4Mut: High, Area ratio for API5 WT/API5 4Mut was about 1).

We confirmed the interaction of API5 with some proteins in the mRNA export machineries by immunoprecipitation (Figure 4C). To rule out the RNA-mediated protein pulldown, the samples were treated with RNase A. Similar to the proteomics results, UAP56 and LRPPRC bound API5 regardless of the mutation in API5, whereas THOC2 and eIF4E bound only to API5 WT. In summary, these data suggest that API5–FGF2 associates with the mRNA export machineries of the nucleus.

### API5 and FGF2 interact directly with UAP56, a common factor of the TREX and eIF4E/LRPPRC mRNA export machineries

Because our immunoprecipitation/proteomics data suggested the presence of API5-binding partners in two different mRNA export pathways, it is plausible that the API5–FGF2 complex associates with a factor common to multiple mRNA export pathways. hnRNPA1, DDX3X, and UAP56 are common to both NXF1- and CRM1-dependent mRNA export pathways (33). Among these, only UAP56 was found to be an API5 binding partner in our immunoprecipitation/proteomics data (Figure 4; Table 2). Moreover, it is known that a plant API5 interacts with DEAD box RNA helicases which share high sequence identities with DDX39A and UAP56 (34). Therefore, we investigated whether API5 or FGF2 interacts with UAP56 directly. GST pulldown and SPR experiments with purified recombinant proteins showed discernible direct interactions between UAP56 and API5 (Figure 4D and E). Unexpectedly, UAP56 also directly interacted with LMW FGF2 with a cooperative interaction among UAP56, API5 and FGF2. The *K*_d_ values determined by SPR for UAP56–API5, UAP56–FGF2 and UAP56–API5/FGF2 were 1.37 (±0.00) μM, 382 (±6) nM and 199 (±3) nM, respectively. Considering the k_on_, k_off_, and t_1/2_ values from the SPR data, the complex formation rate of UAP56–FGF2 is slightly higher than that of UAP56–API5 and protein complex stability was also slightly increased when all three proteins were associated together (Figure 4E; Supplementary Table S6). These data suggest that API5–FGF2 is involved in both NXF1- and CRM1-dependent mRNA export pathways through direct interaction with UAP56.

### API5–FGF2 complex functions in bulk mRNA export

**Figure 5.**
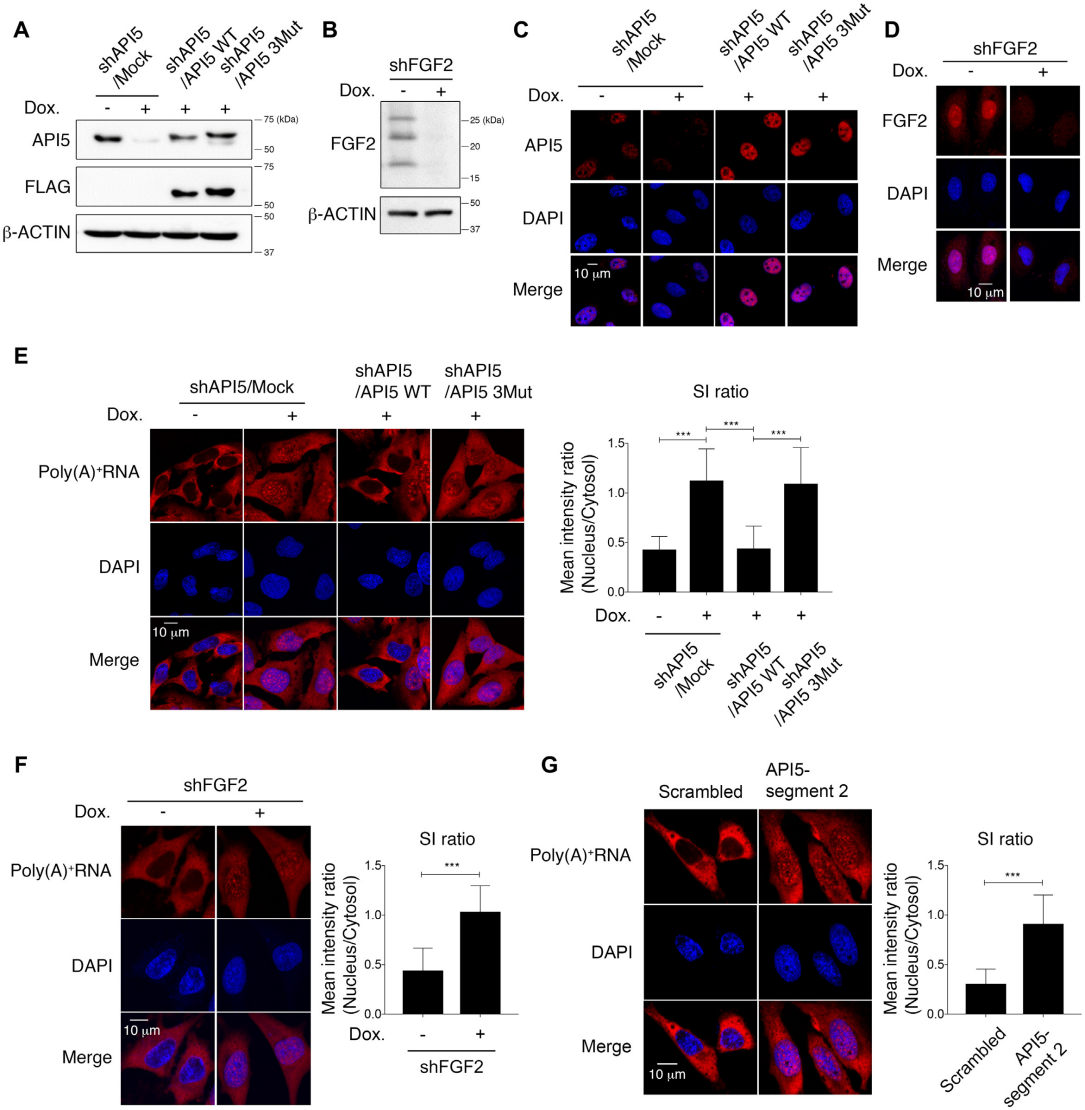
API5 functions in bulk mRNA export. (**A**) Western blot analysis of API5-depleted and API5 WT- and 3Mut-reconstituted HeLa cells. Dox., doxycycline. (**B**) Western blot analysis of FGF2-depleted HeLa cells. β-Actin was used as a loading control. (**C**) Determination of API5 depletion and reconstitution of API5 WT or 3Mut by immunocytochemistry. (**D**) Determination of FGF2 (HMW and LMW FGF2) depletion by immunocytochemistry. (**E**) Bulk mRNA export was monitored by RNA-FISH using an oligo(dT) probe following API5 depletion (shAPI5/Mock) and reconstitution (API5 WT or API5 3Mut). Cells were treated with actinomycin D for 2 h before the FISH experiment to reduce nascent RNA signals. The mean SI ratios of the nuclear/cytosolic fractions were quantified (***P < 0.001, *n* = 40 for each sample). (**F**) Same RNA-FISH experiment with FGF2 depletion (***P < 0.001, *n* = 51 for each sample). (**G**) Same RNA-FISH experiment after disruption of the API5–FGF2 interaction by treatment with lentiviruses expressing API5-segment 2 peptide (***P < 0.001, *n* = 50 for each sample).

To determine the function of API5 and FGF2 related to mRNA export, we developed a doxycycline-induced shRNA expression system in HeLa cells for establishing API5- and FGF2-depleted cell lines (Figure 5A–D). Because the TREX complex controls bulk mRNA export, poly(A)^+^ tail-containing bulk mRNA was monitored in HeLa cells using FISH with an oligo(dT) probe (Figure 5E). While the control cells showed significantly lower levels of nuclear poly(A)^+^ RNA than cytosolic mRNA, API5 depletion by doxycycline-induced shRNA resulted in the accumulation of poly(A)^+^ RNA in the nuclei in speckled patterns, suggesting a role of API5 in mRNA export (Figure 5E). API5 WT reconstitution mitigated this accumulation of bulk mRNA in the nuclei, whereas reconstitution of the API5 mutant which is unable to bind FGF2 (API5 3Mut) did not. Similarly, bulk mRNA export was blocked when FGF2 was depleted (Figure 5F).

In addition to the mutant study, we further confirmed the function of API5–FGF2 complex related to mRNA export through the disruption of the protein complex. We tested whether the API5- and FGF2-derived synthetic peptides (API5-segment 2 and FGF2-segment 1 in Figure 1D) can decrease the API5–FGF2 interaction *in vitro*, and found that the API5-segment 2 more effectively inhibits the API5–FGF2 interaction (Supplementary Figure S9). Assuming that the expression of peptides in cells would not influence the FGF2 signaling initiating from the FGF2–FGFR–heparin association on the cell surface, the lentivirus mediated expression of API5-segment 2 peptide to disrupt the API5–FGF2 interaction in cells were performed. The API5-segment 2 peptide expression in cells were not only effective in directly disrupting the API5–FGF2 interaction in an immunoprecipitation experiment (Supplementary Figure S9), but also in reducing the nuclear export of bulk mRNA (Figure 5G). These data imply that the bulk mRNA export activity of API5 depends largely on its interaction with FGF2.

### API5–FGF2 complex regulates the export of 4E-SE containing oncogenic mRNA

**Figure 6.**
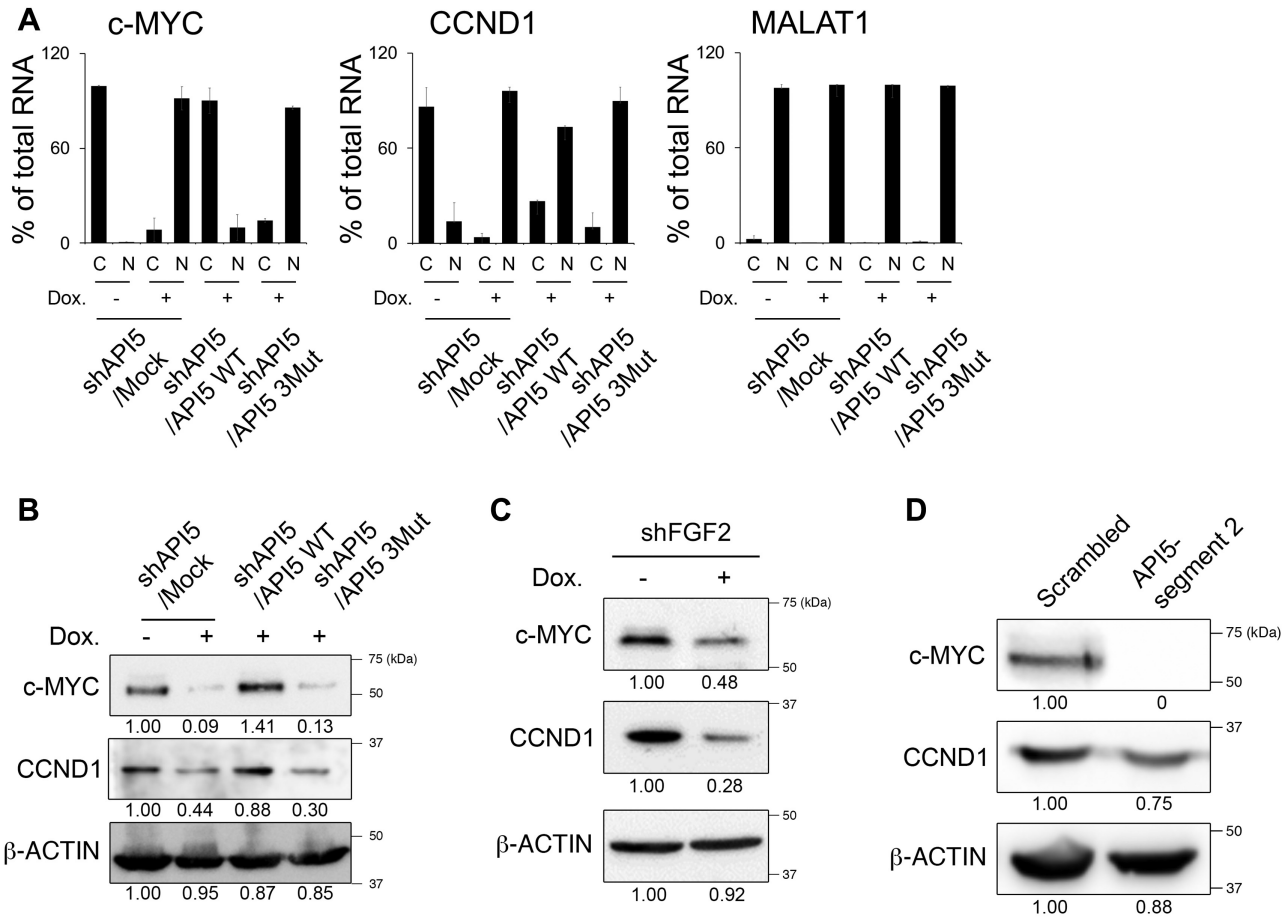
API5 functions in 4E-SE containing mRNA export. (**A**) RT-qPCR analysis of the subcellular levels of 4E-SE-containing mRNAs following API5 depletion and reconstitution. ‘C’ is the cytosolic fraction and ‘N’ is the nucleus fraction. (**B**) Protein levels were monitored by western blot analysis for the protein products of the representative 4E-SE-containing genes c-MYC and cyclin D1 following API5 depletion and reconstitution. (**C**) Western blot analysis showing the c-MYC and cyclin D1 levels following FGF2 depletion. (**D**) Western blot analysis showing the c-MYC and cyclin D1 levels after treatment with lentivirus expressing the API5-segment 2 peptide. Numbers below the western blots indicate the expression of proteins as measured by fold change.

Since API5–FGF2 can interact with the eIF4E/LRPPRC complex in the CRM1-dependent pathway, we investigated whether API5–FGF2 can control the specific mRNAs containing an eIF4E sensitivity element (4E-SE) (32,35). Because of the low signals of RNA FISH for the specific mRNA of a single gene, we performed subcellular fractionation and used RT-qPCR to measure the levels of several 4E–SE-containing mRNAs. Among these, *c-MYC* and cyclin D1 (*CCND1*) levels in the nucleus and cytosol were controlled by the API5–FGF2 interaction. The nuclear mRNA levels of *c-MYC* and *CCND1* were greatly increased when API5 was depleted, implying the accumulation of these mRNAs (Figure 6A). Reconstitution with API5 WT completely reversed the nuclear accumulation of *c-MYC* mRNA, while reconstitution with API5 3Mut did not. *MALAT1* RNA, which is always localized in the nucleus, was not affected by API5 depletion. To investigate whether the inhibition of mRNA export from the nucleus to the cytosol influenced the protein expression of these genes, we monitored the protein levels of c-MYC and cyclin D1 by immunoblotting (Figure 6B). As expected, the expression of these oncogenic proteins was downregulated by API5 depletion and was restored only by API5 WT reconstitution but not by API5 3Mut reconstitution. Knockdown of FGF2, and the disruption of API5–FGF2 interaction by expression of API5-segment 2 also showed reduced protein expression of c-MYC and cyclin D1 (Figures 6C and D). Consistent with these results, HeLa cells treated with the CRM1 inhibitor, selinexor, reduced protein levels of c-MYC and cyclin D1 (Supplementary Figure S10). Similar results were shown in previous reports (36,37), which further suggest that API5-FGF2 functions along the same CRM1 pathway. Taken together, these data imply that API5 affects the CRM1 pathway of the mRNA export in a way that depends on its interaction with FGF2.

## DISCUSSION

In this study, we determined the crystal structure of the API5–FGF2 complex. The structural superposition of the API5–FGF2 and FGF2–FGFR1–heparin complexes with FGF2 as the reference revealed that steric clashes would occur if API5 instead of heparin binds to the FGF2 bound FGFR dimer, implying that API5 is unlikely to affect the role of the FGF2 bound FGFR dimer on the extracellular surface (Supplementary Figure S5). Because API5 and HSPGs binds to similar residues of FGF2, API5 may stabilize FGF2 in the nucleus as HSPGs do on the cell surface. Although FGF2–heparin interaction was relatively strong, probably because it forms a stable complex to protect FGF2 from proteolysis and thermal denaturation on the cell surface (Supplementary Figure S6), the API5–FGF2 interaction was weaker than the FGF2–heparin interaction. This suggests possible dynamic changes in this large protein complex in the nucleus (Figure 1). However, since FGFR1 is sometimes found in the nucleus inducing cell proliferation and invasion (38,39), the association of API5, FGF2, and FGFR1 in the nucleus cannot be fully excluded.

LMW FGF2 has been reported to localize in the nucleus by a cryptic NLS (18). However, the underlying mechanism has not yet been revealed. In this study, we found that the API5-interacting region of FGF2 overlaps with its previously suggested cryptic NLS region, which provided important insight into the molecular mechanism of its nuclear localization. The fact that LMW FGF2 localized mainly in the nucleus when co-expressed with API5, but in the cytoplasm if it fails to bind API5 suggests that API5 acts as a carrier protein for FGF2 trafficking to the nucleus (Figure 3). However, still some LMW FGF2 was found in the nucleus even without binding to API5, hence the presence of additional mechanisms cannot be ruled out.

Clues on the role of API5 in mRNA export machinery have been implied in various reports. For instance, Influenza virus A nucleoprotein, an API5 interacting protein that contributes to the negative regulation of API5 functions and enables viral replication (15), interacts with UAP56 and ALYREF (40,41). In addition, the interaction between API5 and UAP56 homologs in plants (34) suggests a direct interaction between API5 and UAP56 in human cells despite the fact that API5 does not contain a UAP56 binding motif which is usually found in other TREX component proteins such as ALYREF, UIF, CHTOP and LUZP4 (42). In this study, we found that API5 interacts with UAP56 not only in cells but also at the level of purified proteins (Figure 4), which suggests that API5 and UAP56 interact directly in physiological conditions.

Among the many proteins in the mRNA export machineries, UAP56 is a critical player. UAP56 interacts with various TREX components, and together with ALYREF, UAP56 is an essential for cell viability (27,42). Moreover, UAP56 depletion results in a severe mRNA export defect in human cells (27). Notably, UAP56 is one of the factors common in both TREX and eIF4E/LRPPRC export machineries (33), which explains why API5–FGF2 is possibly involved in both the TREX and eIF4E/LRPPRC complexes. In our immunoprecipitation result, while UAP56 and LRPPRC interacted with API5 regardless of the FGF2 interaction, some API5-binding partners, such as THOC2 and eIF4E, interacted with API5 in a FGF2-dependent manner (Figure 4C). Moreover, the cellular phenotypes on mRNA export were also dependent on the API5–FGF2 interaction (Figures 5 and 6), further suggesting that the complex formation of API5 and FGF2 is required for the function of the TREX or eIF4E/LRPPRC mRNA export machineries.

Many mRNA export-related proteins are dysregulated in cancers (31,43). For instance, the eIF4E/LRPPRC-mediated CRM1 pathway is upregulated in many cancers (43). The eIF4E and LRPPRC in the CRM1 pathway were API5-interacting partners in our interactome analysis. The CRM1 pathway governs the selective export of various mRNAs involved in cell proliferation and survival, such as cyclin B1, cyclin D1, cyclin E1, NBS1, PIM1, ODC, c-MYC and MDM2 (35). Recently, the CRM1 inhibitor selinexor (44,45) has been approved by the FDA for the treatment of multiple myeloma, and the eIF4E inhibitor ribavirin has been shown to induce regression of aggressive B-cell lymphomas (46), both supporting the usability of cancer therapeutics targeting the CRM1 pathway. It has been known that eIF4E controls c-MYC translation (47) as well as mRNA export (35), and targeting eIF4E can regulate c-MYC both at the mRNA export and translation level. Similarly, since we observed the decrease of mRNA export and of protein expression of c-MYC and cyclin D1 by disruption of the API5−FGF2 complex formation (Figure 6), the API5−FGF2 interaction can be an additional potential therapeutic target for cancers. In this context, the three-dimensional structure of the API5−FGF2 complex can be further exploited for protein-protein interaction inhibitor discovery.

**Figure 7.**
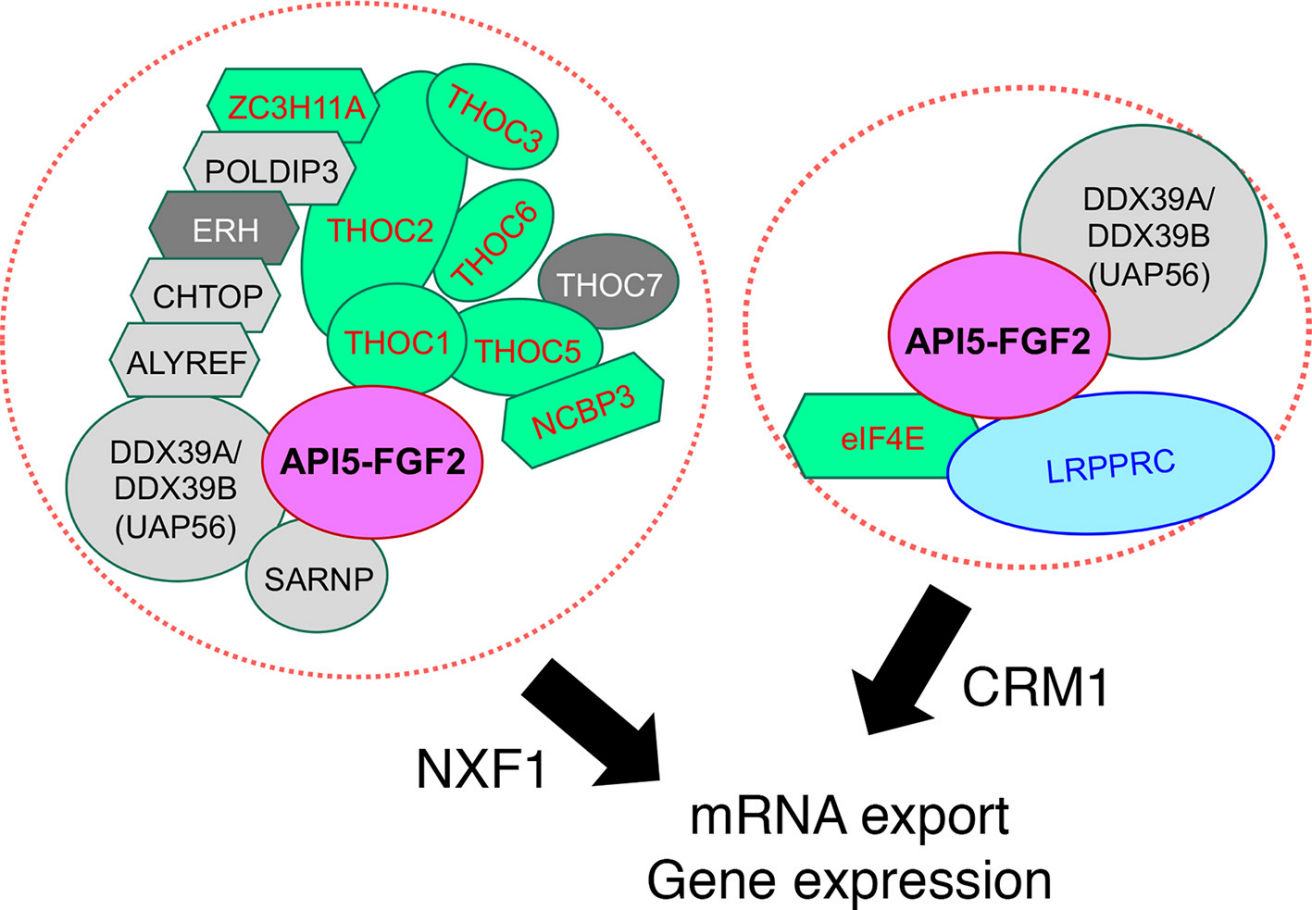
Model of the API5–FGF2 function A model showing the function of API5–FGF2 in mRNA export and gene expression.

Although the nuclear localization of FGF2 has long been observed, the detailed molecular function of nuclear FGF2 has not yet been demonstrated. Our results provide new insight into how the nuclear form of FGF2 functions with API5 in the nucleus and suggest a mode of anti-apoptotic function distinct from that of canonical inhibitors of apoptosis proteins (IAP). To conclude, our study points to a model for the function of the API5-FGF2 complex in the TREX and eIF4E/LRPPRC mRNA export pathways (Figure 7).

## DATA AVAILABILITY

Atomic coordinates and structural factors have been deposited in the Protein Data Bank (PDB) under accession number 6L4O. The mass spectrometry proteomics data have been deposited to the ProteomeXchange Consortium via the PRIDE partner repository with the dataset identifier PXD013517 (48).

## Supplementary Material

gkaa335_Supplemental_File
