# Supplementary material for: Regulation of mRNA export through API5 and nuclear FGF2 interaction
Source: Nucleic Acids Res. 2020 May 8;48(11):6340–52. doi: 10.1093/nar/gkaa335 (PMC7293033; doi:10.1093/nar/gkaa335)
Supplement: gkaa335_Supplemental_File [file gkaa335_supplemental_file.pdf]

Supplementary Information for

Regulation of mRNA export through API5 and nuclear FGF2 interaction

Seoung Min Bong[1,†], Seung-Hyun Bae[1,2,†], Bomin Song[1], HyeRan Gwak[1], Seung-Won Yang[1], Sunshin Kim[1], Seungyoon Nam[3], Krishnaraj Rajalingam[4], Se Jin Oh[5], Tae Woo Kim[5], SangYoun Park[6], Hyonchol Jang[1,2,*], Byung Il Lee[1,2,*]

[1]Research Institute, National Cancer Center, Goyang-si, Gyeonggi 10408, Republic of Korea

[2]Department of Cancer Biomedical Science, National Cancer Center Graduate School of Cancer Science and Policy, Goyang-si, Gyeonggi 10408, Republic of Korea

[3]Department of Life Sciences, College of BioNano Technology and Department of Genome Medicine and Science, Graduate School of Medicine, Gachon University, Incheon 21565, Republic of Korea

[4]Cell Biology Unit, University Medical Center Mainz, JGU, Mainz, Germany

[5]Department of Biomedical Sciences, Graduate School of Medicine, Korea University, Seoul 02841, Republic of Korea.

[6]School of Systems Biomedical Science, Soongsil University, Seoul 06978, Republic of Korea

[†]These authors contributed equally to this work

*To whom correspondence should be addressed: Tel: +82-31-920-2223; Email: bilee@ncc.re.kr

Correspondence may also be addressed to: Tel: +82-31-920-2239; Email: hjang@ncc.re.kr

Supplementary Materials and Methods

GST pulldown assays and SPR analysis

To test the direct interactions between the proteins, GST pulldown and SPR experiments with highly purified proteins were conducted. The same amount of full-length GST-API5 WT, GST-API5 3Mut (E184A/D185A/E190A), and GST proteins (approximately 4.6 nmole each) was used for pulldown assays. Purified FGF2 (LMW FGF2; residues 135–288; C211S/C229S mutant; where C211S/C229S mutation was introduced for the expression of recombinant FGF2 in *E. coli*) and FGF2 Mut (same construct, C211S/C229S/R262A/T263A/K271A mutant) were also prepared. Purified GST-UAP56 was used for monitoring the interaction among UAP56, API5, and FGF2. Glutathione-agarose resin (GE Healthcare, USA) was used for GST pulldown assays.

Protein-protein interactions were also investigated by SPR spectrometry (SR7000DC, Reichert, USA). To immobilize the proteins, purified recombinant API5 WT, API5 3Mut, or UAP56 protein was passed over the chip surface. Each protein was immobilized through standard amino acid coupling on a carboxymethyl dextran hydrogel surface sensor chip (Reichert, USA) until saturation was achieved. Recombinant PBK (PDZ-binding kinase) was used as a negative control for the UAP56 interaction. A reference flow cell was used to record the response for bovine serum albumin (BSA) as a control, and the value of the response for BSA was subtracted from the value of the response for each sample. To monitor the interaction between FGF2 and heparin, heparin oligosaccharide dp10 (Iduron, UK) was used. SPR data were fitted using Scrubber2 software (BioNavis, Finland). The equilibrium dissociation rate constant (K_d) was calculated using the equation: $K_d = k_{off} / k_{on}$ where the k_{on} is the association rate constant ($M^{-1}s^{-1}$) and k_{off} is the dissociation rate constant (s^{-1}). The half-lives of the complexes were estimated using the equation: $t_{1/2} = \ln 2 / k_{off}$.

Generation of *API5* knockout/knockdown cells

All experiments were carried out in accordance with protocols approved by the National Cancer Center Institutional Biosafety Committee (approval number 17-NCCIBC-039). HeLa cells were purchased from the American Type Culture Collection (ATCC) and cultured in MEM (HyClone, USA) supplemented with 10% heat-inactivated fetal bovine serum (FBS) (HyClone, USA), 1% penicillin–streptomycin, and 5 μg/ml Cellmaxin plus (GenDEPOT, USA) at 37°C in a humidified 5% CO_2 atmosphere. In addition, 293FT cells (Thermo Fisher Scientific, USA) were cultured in DMEM (HyClone, USA) supplemented

with 10% heat-inactivated FBS and 1% penicillin–streptomycin. Lentiviruses were produced according to a method described previously(1). Briefly, 293FT cells were co-transfected with Tet-pLKO or lentiCRISPR v2 vectors and packaging vectors (psPAX2 and pMD2.G, a gift from Didier Trono, Addgene plasmid # 12260 and Addgene plasmid # 12259, respectively) using polyethylenimine (Polysciences Inc., USA). HeLa cells were infected with filtered lentiviruses in the presence of 0.8 μg/ml polybrene (Sigma, USA) for 5 hours. Two days after infection, the infected cells were selected with 2 μg/ml puromycin (Amresco, USA) for at least additional 4 days. The *API5* knockout cells generated by the CRISPR/Cas9 system were named HeLa gAPI5, and the *API5* knockdown cells generated by the Tet-pLKO system were named HeLa Tet-on-shAPI5. For *API5* knockdown using the Tet-pLKO system, doxycycline (1 μg/ml, Sigma-Aldrich, USA) was added every two days.

Generation of API5-reconstituted cells

API5 knockout HeLa cells (HeLa gAPI5) were reconstituted with Mock, API5 WT or API5 mutant by transfection with empty vector (pCAG-F-BS), pCAG-F-API5 WT vector or 4Mut (D145A/E184A/D185A/E190A) vector, respectively, using Lipofectamine 3000 (Life Technologies, USA). Transfected cells were selected with blasticidin (10 μg/ml, Invitrogen, USA) for at least 7 days. Because the guide RNAs were designed to target the region spanning both the exon and the intron, exogenously added constructs were not targeted by the CRISPR/Cas9 system. The corresponding API5-reconstituted knockout cells were named gAPI5/Mock, gAPI5/API5 WT and gAPI5/API5 4Mut. For API5 reconstitution in conditional knockdown cells, HeLa cells (HeLa Tet-on-shAPI5) were transfected with pCAG-F-Mock, pCAG-F-API5 WT-R, or pCAG-F-API5 3Mut-R, which were generated by site-directed mutagenesis of pCAG-F-API5 WT and 3Mut to introduce mutations conferring resistance to shAPI5 (mutation of nt 939–944 from acaagg to acGGgg). The API5-reconstituted conditional knockdown cells were correspondingly named shAPI5/Mock, shAPI5/API5 WT, and shAPI5/API5 3Mut.

Lentiviral co-expression of peptides and GFP

API5-derived segment and scrambled segment peptides were cloned into pUltra (Addgene #24129). In the pUltra system, GFP and peptides were co-expressed because they are linked by P2A self-cleaving peptide sequences. Lentivirus was produced in the same way as Tet-pLKO.

Immunoprecipitation, proteomics, and pathway analyses

For the purification of API5-binding proteins, HeLa gAPI5/Mock, gAPI5/API5 WT, and gAPI5/API5 4Mut cells were lysed with IP150 buffer (150 mM NaCl, 25 mM Tris 8.0, 0.1% NP-40, 10% glycerol, and 1 mM EDTA), sonicated mildly and centrifuged. Cell extracts were subjected to immunoprecipitation using an anti-FLAG M2 affinity gel (Sigma-Aldrich, USA). Bound proteins were eluted with 3× FLAG peptide (0.1 mg/ml, Sigma-Aldrich, USA). Each eluate was treated with trypsin, and samples were analyzed using a Q Exactive Quadrupole Orbitrap Mass Spectrometer (Thermo Scientific, USA) at the Research Core Center (National Cancer Center Korea). Functional analyses were performed using Ingenuity Pathway Analysis (IPA) software(2) (Qiagen, Germany). The UniProt gene identifiers of API5 interaction partners were uploaded into the software, and core analyses were performed to identify functions and pathways. For the immunoprecipitation experiment validating the interactions between API5 and mRNA export complexes, RNase A-treated samples were used to rule out RNA-mediated interactions.

Immunoblotting, immunofluorescence, and antibodies

Immunoblotting was performed as described previously(1). Immunoblot images were obtained using Fusion SL/SOLO imaging system (Vilber Lourmat, France). Selinexor (#S7252) was purchased from Selleckchem. Immunofluorescence staining was performed as described previously(3). Confocal imaging was performed at the Research Core Center (National Cancer Center Korea) on an LSM780 system (Carl Zeiss, Germany).

Supplementary Figures

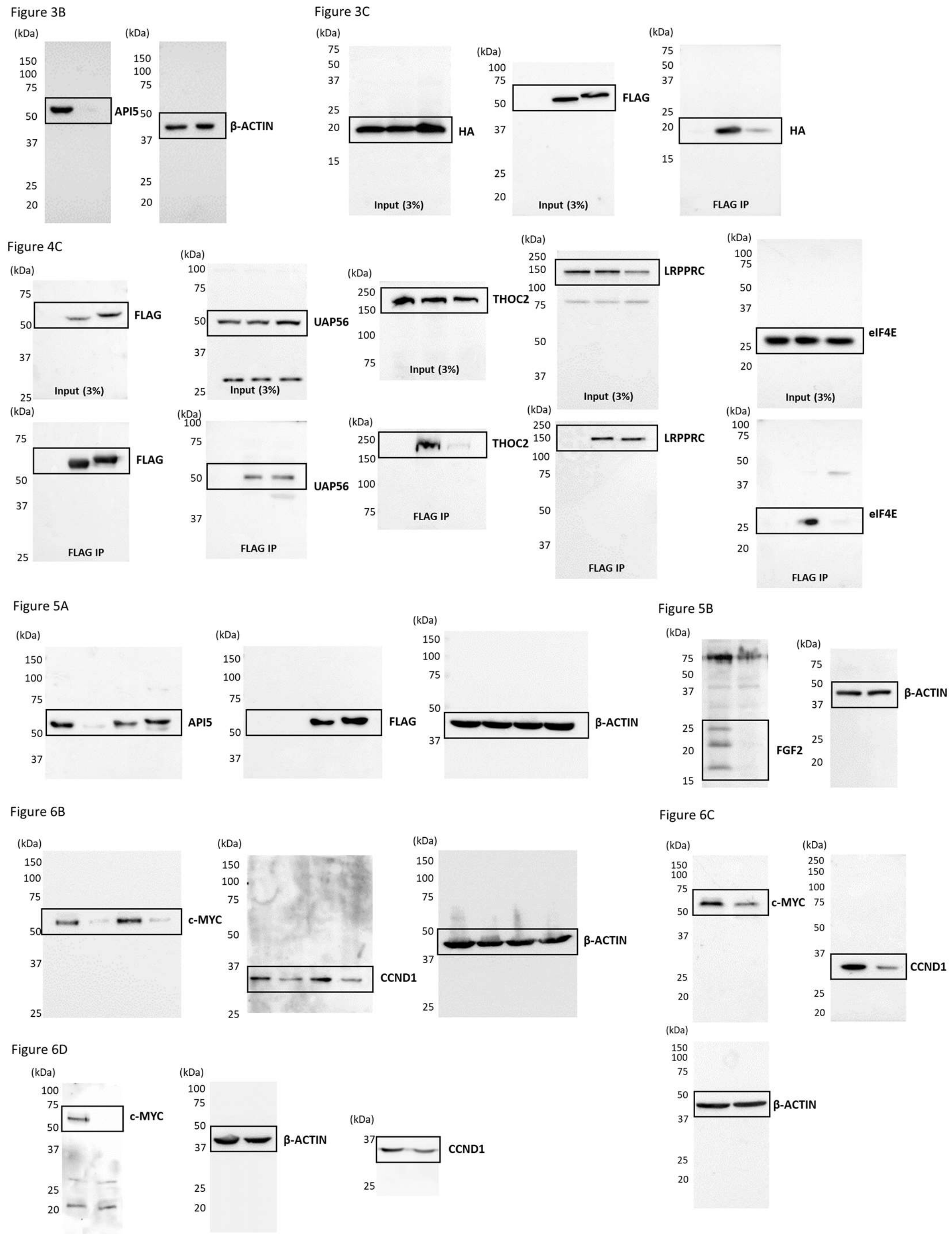

Figure S1. Full immunoblots. Full-length images of western blot analyses.

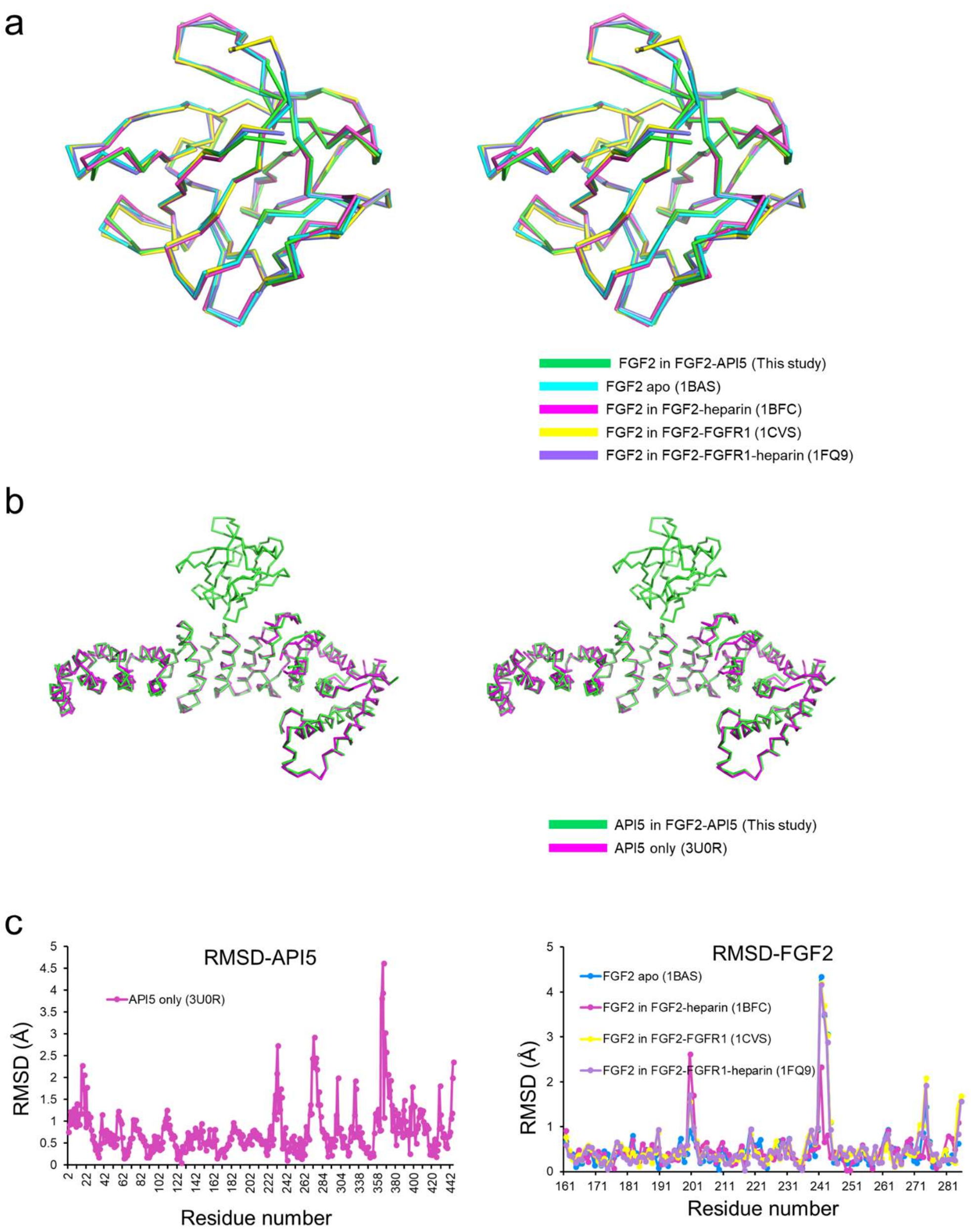

Figure S2. Stereoview of the structural superposition of API5 and FGF2 in the API5–FGF2 complex with other FGF2 or API5 structures. The PDB entries are shown in parentheses. (**a**) Comparison of FGF2 structure with other previously determined FGF2 structures, including FGF2 apo, the FGF2–heparin complex, the FGF2–FGFR1 complex, and the FGF2–FGFR1–heparin ternary complex structures. (**b**) Comparison of the API5 structure in the FGF2–API5 complex to the API5-only structure. (**c**) The r.m.s.d. plot for the structural comparison of the API5 structure in the API5–FGF2 complex to the API5-only structure (left) and the FGF2 structure in the API5–FGF2 complex to other FGF2 structures (right). The average r.m.s.d. between the API5 structure in the API5–FGF2 complex (6L4O) and the API5-only structure (3U0R) was 0.972 Å. The average r.m.s.d. between the FGF2 structure in the API5-FGF2 complex and other FGF2 structures was 0.579–0.804 Å (0.754 Å against 1BAS, 0.579 Å against 1BFC, 0.804 Å against 1CVS, and 0.778 Å against 1FQ9).

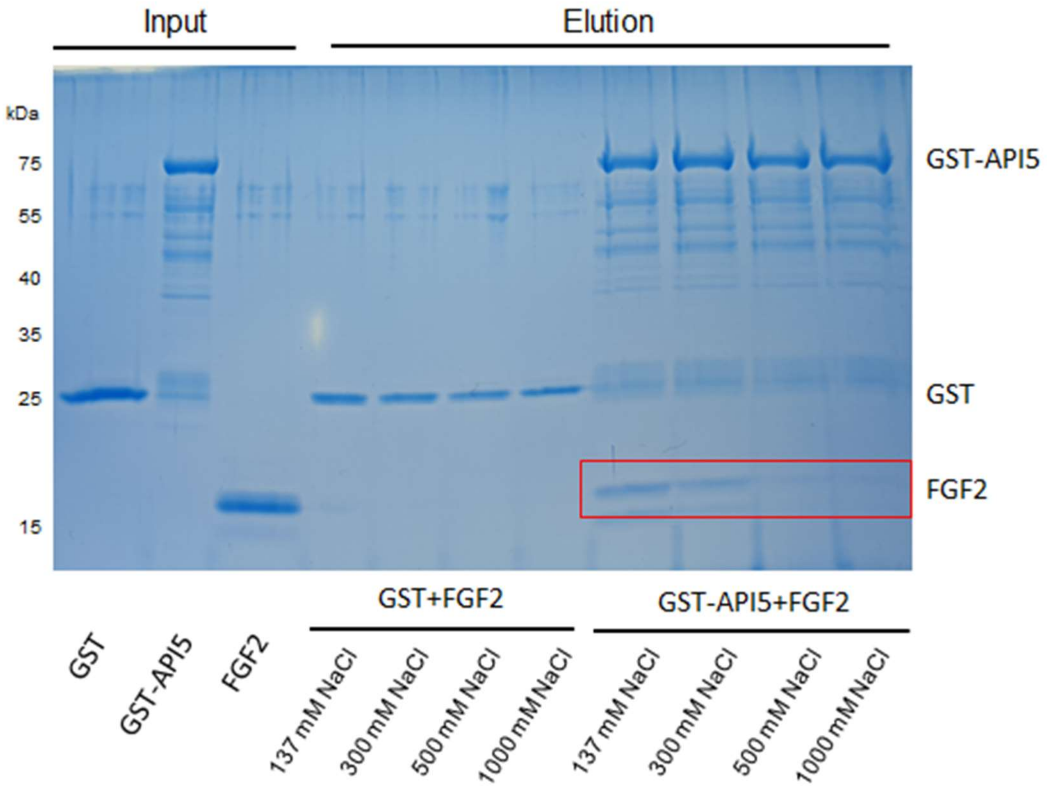

Figure S3. Salt-dependent formation of API5–FGF2 complex. GST pulldown with purified API5 and FGF2 under various salt concentrations (Phosphate buffer pH 7.4 supplemented with 137, 300, 500, and 1000 mM NaCl concentration, respectively). FGF2 bands, pulled down by GST-API5, are shown in red box.

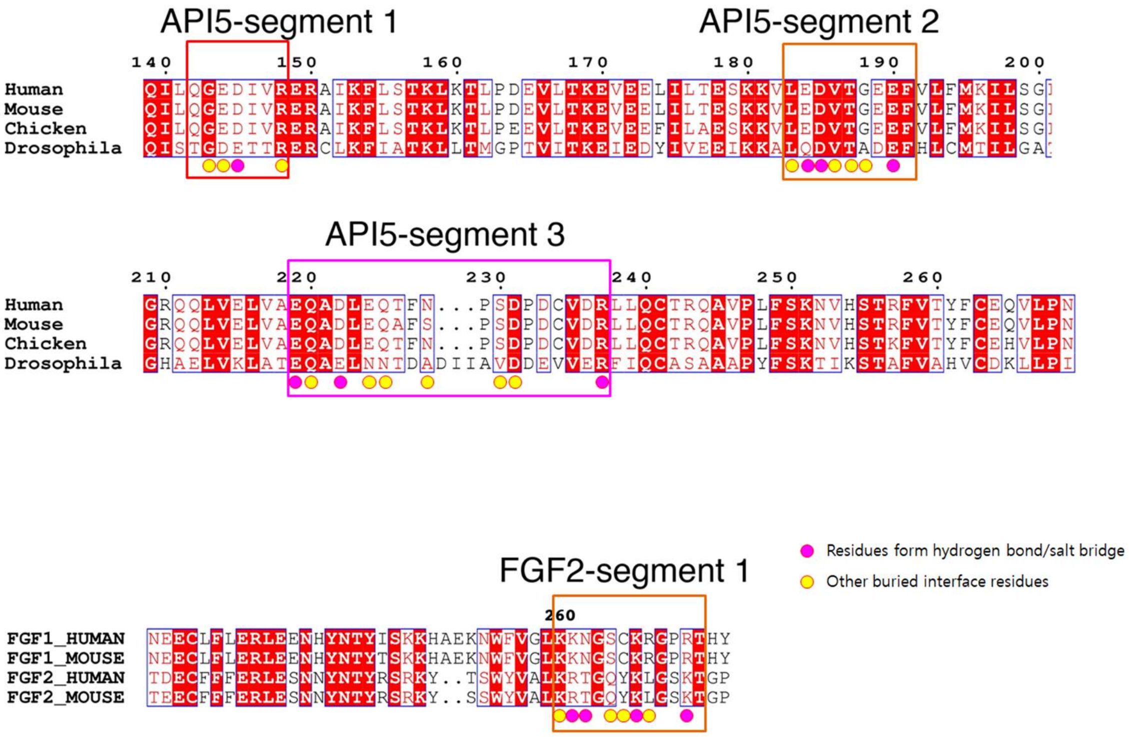

Figure S4. Sequence alignments of amino acid segments on API5 and FGF2 that are involved in protein-protein interactions. Among the 22 FGFs, amino acid sequence alignment was done with FGF1 and FGF2 from human and mouse. Program ClustalW (https://embnet.vital-it.ch/software/ClustalW.html) and Espript3 (http://espript.ibcp.fr/ESPript/ESPript/) were used for this figure(4,5). The residues in blue boxes are strictly or highly conserved residues. The residues involved in hydrogen bond or salt bridge are marked in pink circles and other interface residues are marked in yellow circles. Each segment is shown in red, orange, or pink box, respectively.

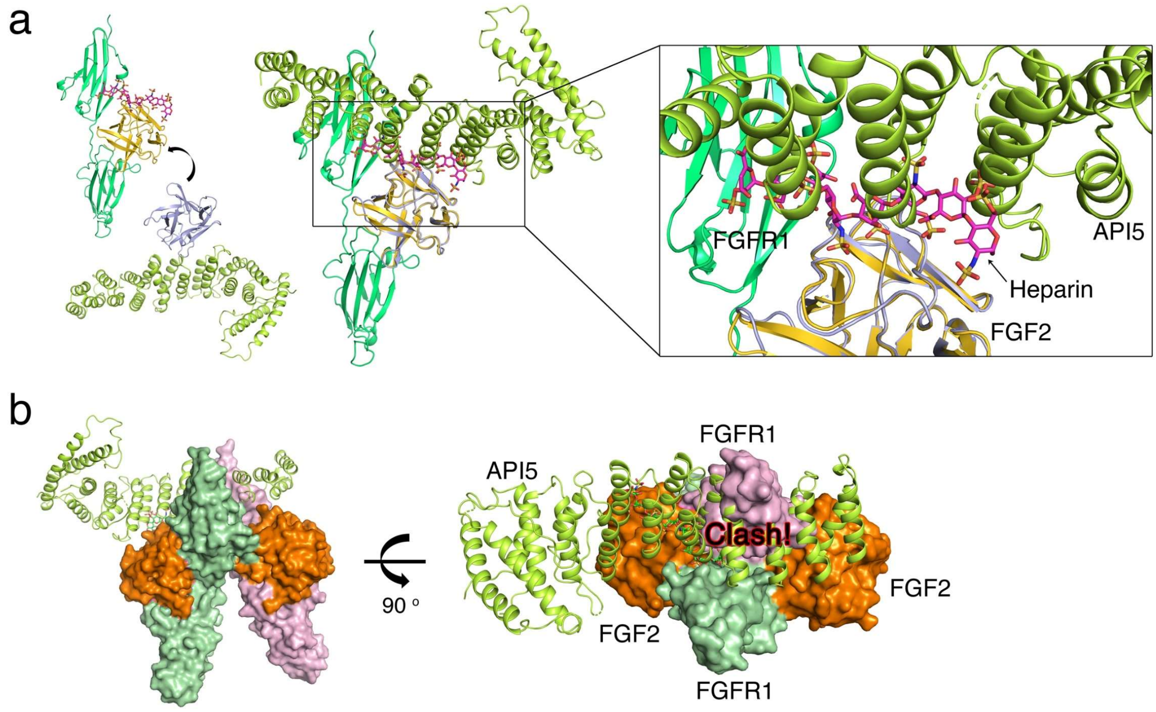

Figure S5. Comparison of the API5–FGF2 complex structure with the FGF2–FGFR1–heparin ternary complex structure. (**a**) Structural superposition of the API5–FGF2 and FGF2–FGFR1–heparin complexes (left). The FGF2 structures were the foci of the superpositions. Detailed view of the superposed structure (right). The heparin molecule in FGF2–FGFR1–heparin complex (PDB entry: 1FQ9) was colored in magenta and API5 from API5–FGF2 complex (PDB entry: 6L4O) was drawn in limon. FGFR1 is drawn in green. FGF2 in FGF2–FGFR1–heparin complex is drawn in yellow orange and FGF2 in API5–FGF2 complex is drawn in light blue. (**b**) API5–FGF2 complex superposed with the FGF2–FGFR1 dimer with FGF2 as the central figure. A structural clash with the FGFR1 molecule is shown. Two FGF2 molecules were colored in orange and FGFR1 molecules were colored in lime green or light pink. The figure was drawn using the program Pymol (Schrödinger LLC, USA).

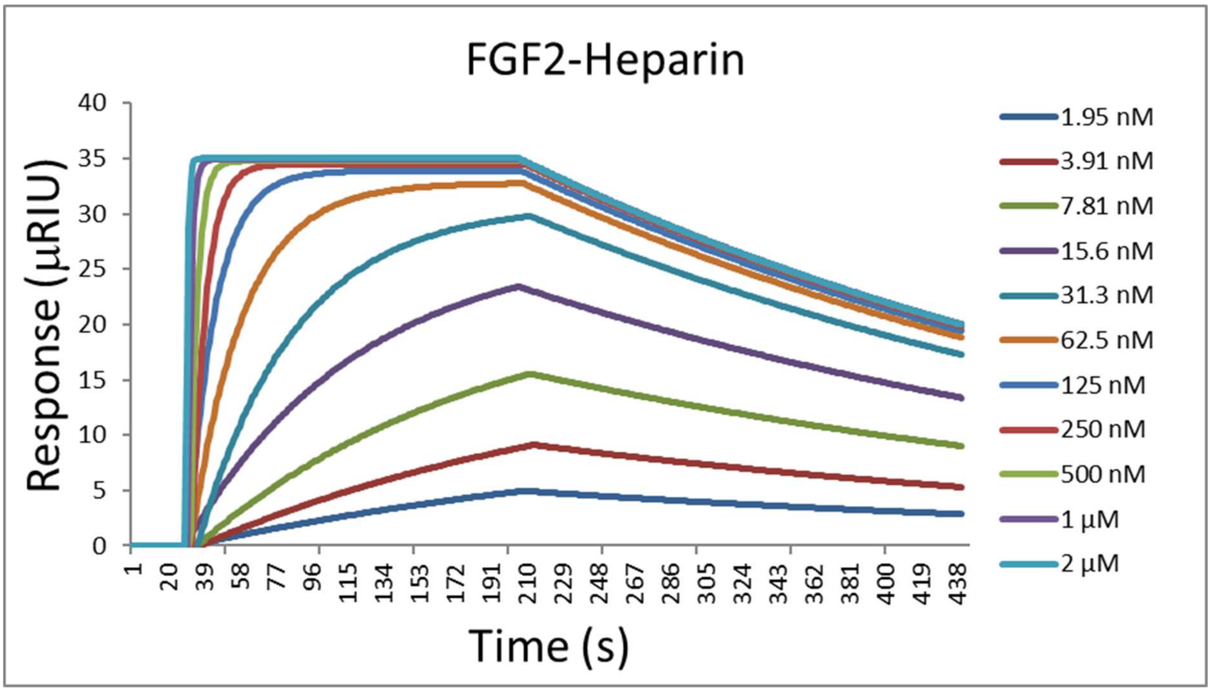

Figure S6. The FGF2 and heparin (heparin oligosaccharide dp10, Induron, UK) interaction was monitored by SPR. The K_d value for heparin binding was approximately 4.4(±0.5) nM. The association (k_{on}) and dissociation rates (k_{off}) were 5.45(±0.06)×10^5 $M^{-1}s^{-1}$ and 2.38(±2) ×10^{-3} s^{-1}, respectively. The calculated half-life of the FGF2-heparin complex was 291 s. SPR experiment was performed with Reichert SR7000DC instrument (Reichert, USA).

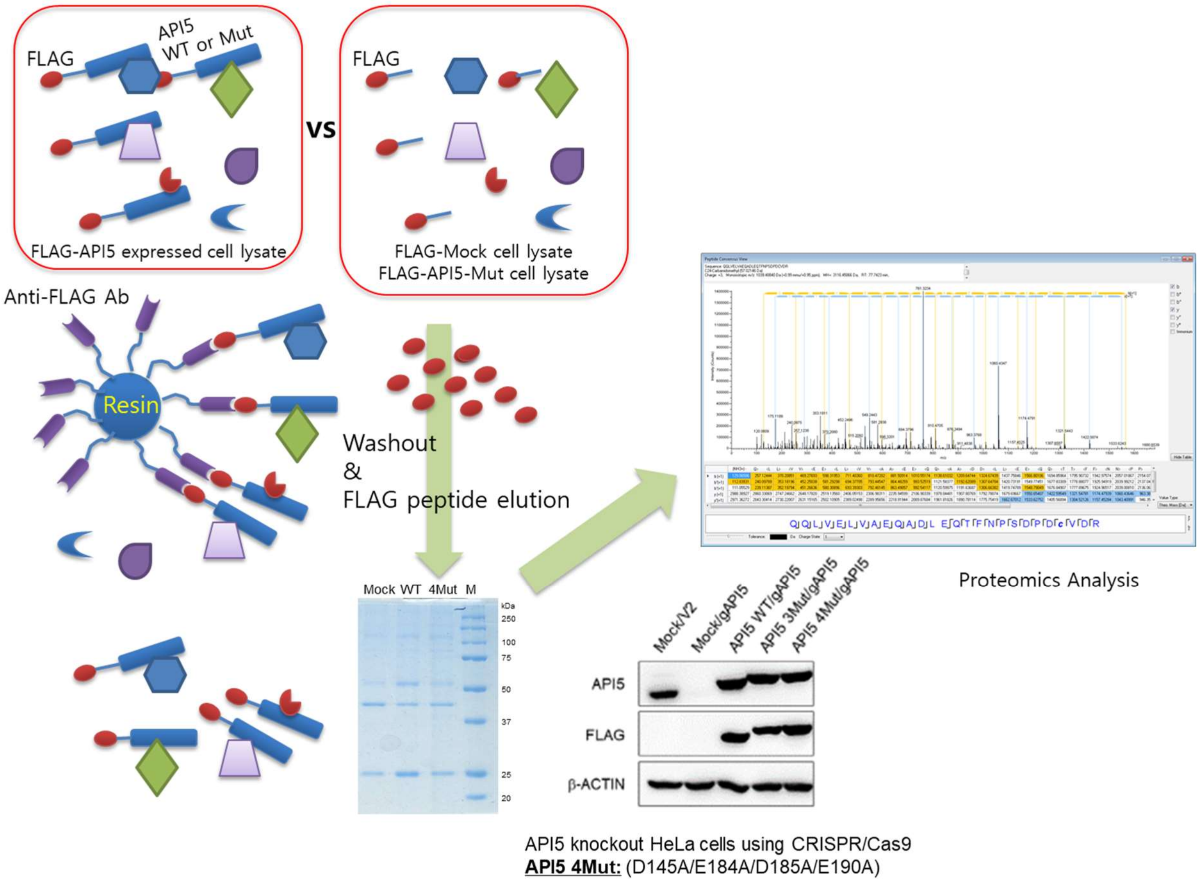

Figure S7. Schematic representation of the method for exploring the API5 interactome by proteomics analysis. Bound proteins were eluted with 3× FLAG peptide (Sigma-Aldrich, USA) to reduce contaminant proteins due to the nonspecific interaction with resin. Q Exactive Quadrupole Orbitrap Mass Spectrometer (Thermo Fisher Scientific, USA) instrument was used for proteomics analysis. The *API5* knockout HeLa cells (gAPI5) were transfected with Mock, API5 wild type (WT), or API5 4Mut (D145A/E184A/D185A/E190A which lacks FGF2 interaction) plasmids and used for immunoprecipitation/proteomics study. Western blot validation of HeLa cells after *API5* knockout (gAPI5) by the CRISPR/Cas9 system and after reconstitution by API5 WT, 3Mut (E184A/D185A/E190A), and 4Mut is shown.

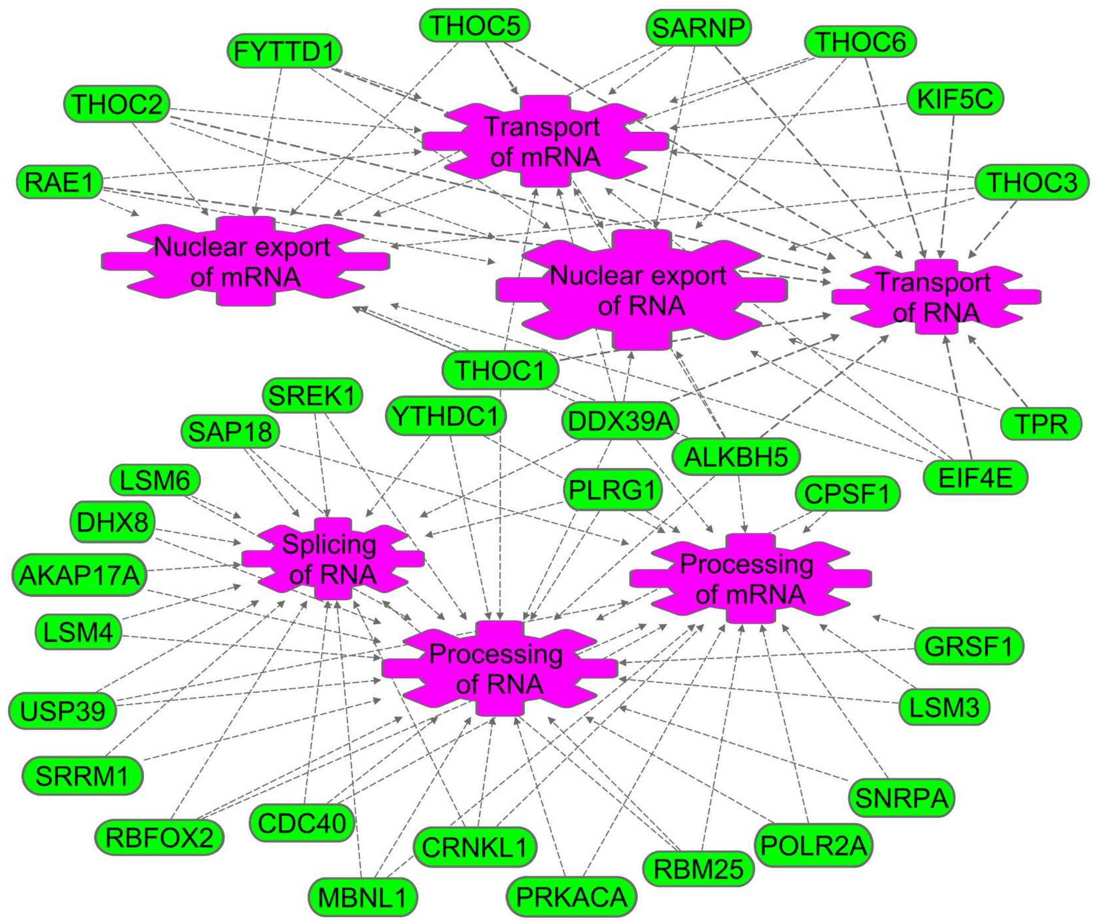

Figure S8. The detailed pathways with the seven lowest *p*-values ($p < 10^{-9}$) in Figure 4A. The program IPA (Qiagen Bioinformatics) was used for generation of this figure. Functional annotations of pathways are shown in magenta and proteins related to each pathway are shown in green.

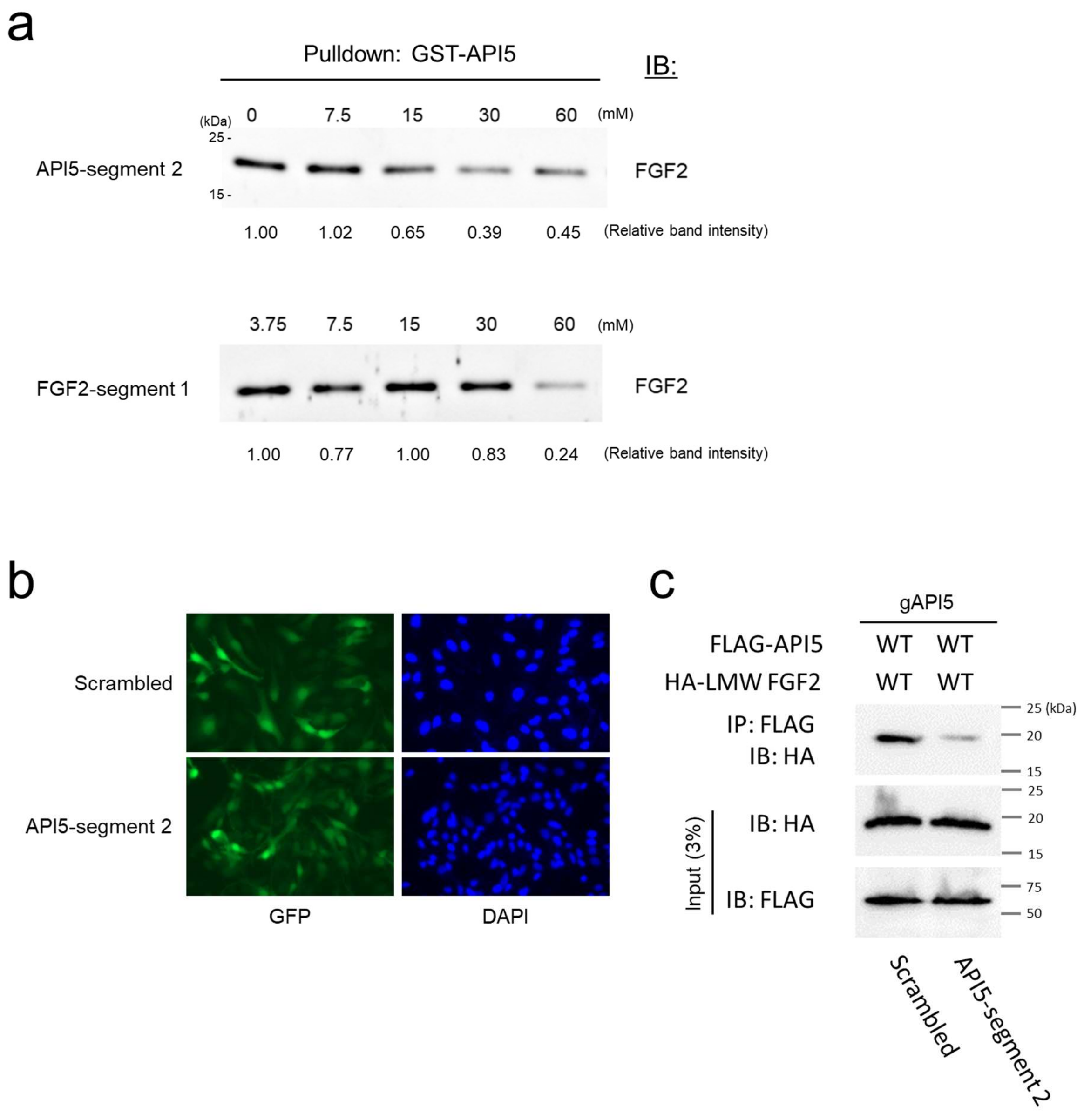

Figure S9. Monitoring the disruption of the API5–FGF2 interaction by API5- and FGF2-derived peptides. (**a**) GST pulldown experiment using GST-API5 and His-FGF2. The amount of FGF2 pulled down was decreased by the addition of the FGF2- or API5-derived peptides. The amino acid sequences for API5 or FGF2-derived peptide segments are shown in Figure 1D. Numbers above the western blots indicate the concentrations of peptide treatment and numbers below the western blots are relative band intensities. (**b**) Monitoring peptide expression levels by lentiviruses. The peptides were expressed in similar levels. Because GFP and peptides are expressed together linked by a P2A self-cleaving peptide sequences, the efficiencies of lentiviral infection and the relative peptide expression levels were determined by monitoring GFP signals using an Axio Imager M2 fluorescence microscope system (Carl Zeiss, Germany). (**c**) Disruption of the interaction between API5 and FGF2 upon API5-segment 2 expression. For the immunoprecipitation experiment, FLAG-API5 and HA-LMW FGF2 were co-transfected and the lentivirus expressing API5-segment 2 peptide was treated to the HeLa cells.

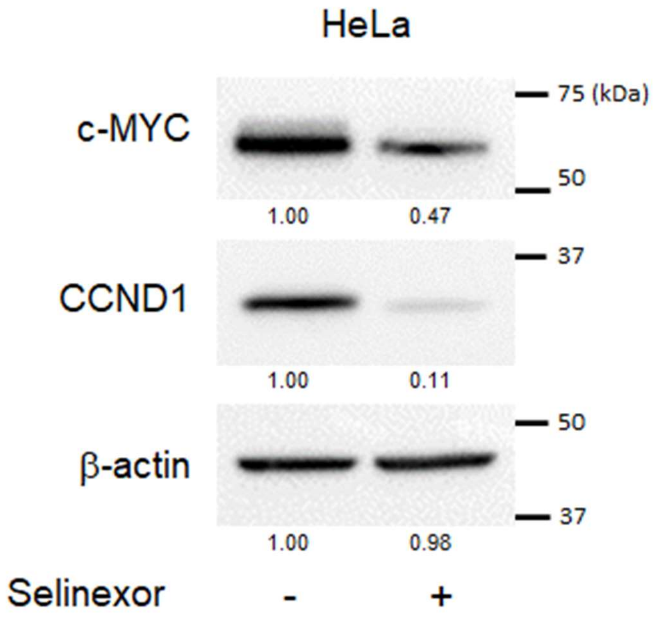

Figure S10. Western blot analysis showing the c-MYC and cyclin D1 levels after treatment with a CRM1 inhibitor. Selinexor (KPT-330) was treated for 72 hours (5 μM final concentration) to the HeLa cells. Numbers below the western blots indicate the expression of proteins as measured by fold change.

Supplementary Tables

Table S1. Plasmids and synthetic gene sequences used in this work

Plasmid name	Plasmid	Description	Tag(s)	Experiments
pET28b-API5	pET-28b(+)	Full-length isoform 2 (1–504), NdeI/NotI	N-6× His	Crystal structure, SPR
pHis-FGF2	Modified pET28	LMW FGF2 (135–288) (C211S/C229S), BamHI/EcoRI (one internal BamHI site exists in synthetic FGF2 gene)	N-6× His	Crystal structure, SPR, Pulldown
pGEX-API5	pGEX-4T-3	Full-length (1–504), EcoRI/NotI	N-GST	Pulldown
pGEX-UAP56	pGEX-4T-3	44–428, BamHI/XhoI	N-GST	Pulldown, SPR
pCAG-F-API5	pCAG-F-BS	Full-length (1–504), XmaI/NotI	N-FLAG	Cell studies
pCAG-HA-LMW FGF2	pCAG-HA-puro	LMW FGF2 (135–288) wild type XhoI/EcoRI	N-HA	Cell studies
ShScramble	Tet-pLKO-puro	5’-CCTAAGGTTAAGTCGCCCTCG-3’		Cell studies
shAPI5 #1	Tet-pLKO-puro	5’-CCACAAGGTTTGTGACATATT-3’		Cell studies
shAPI5 #2	Tet-pLKO-puro	5’-GCAGCAATTTGGGCAACTTTA-3’		Cell studies
shFGF2 #1	Tet-pLKO-puro	5’- GAAACGAACTGGGCAGTATAA-3’		Cell studies
shFGF2 #2	Tet-pLKO-puro	5’- TGAACGATTGGAATCTAATAA-3’		Cell studies
API5-CRISPR	lentiCRISPR v2	5’-CACCGTGTTTTGCAGGGACTTTAGG-3’		Cell studies
pUltra-Scramble	pUltra	5'-GACCGGCAGCTGCAGCTGTCCACCCTGCAGCGGATGCTG-3'		Cell studies
pUltra-API5 segment 2	pUltra	5'-TTGGAGGATGTGACAGGCGAGGAGTTC-3'		Cell studies

>FGF2 (135-288; C211S/C229S)
GCAGCCGGGAGCATCACCACGCTGCCCGCCTTGCCCGAGGATGGCGGCAGCGGCGCCTTCCCGCCCGGCCACTTCAAG
GACCCCAAGCGGCTGTACTGCAAAAACGGGGGCTTCTTCCTGCGCATCCACCCCGACGGCCGAGTTGACGGGGTCCGGG
AGAAGAGCGACCCTCACATCAAGCTACAACTTCAAGCAGAAGAGAGAGGAGTTGTGTCTATCAAAGGAGTGTCAGCTAAC
CGTTACCTGGCTATGAAGGAAGATGGAAGATTACTGGCTTCTAAATCTGTTACGGATGAGTGTTTCTTTTTTGAACGATTGG
AATCTAATAACTACAATACTTACCGGTCAAGGAAATACACCAGTTGGTATGTGGCACTGAAACGAACTGGGCAGTATAAACT
TGGATCCAAAACAGGACCTGGGCAGAAAGCTATACTTTTTCTTCCAATGTCTGCTAAGAGCTGA

>FGF2 (135-288; C211S and C229S are colored in red in *E. coli* expressed recombinant protein)
^{135}AAGSITTLPALPEDGGSGAFPPGHFKDPKRLYCKNGGFFLRIHPDGRVDGVREKSDPHIKLQLQAEERGVVSIKGV**S**ANRYLA
MKEDGRLLASK**S**VTDECFFFERLESNNYNTYRSRKYTSWYVALKRTGQYKLGSKTGPGQKAILFLPMSAKS^{288}

Mutant information

API5 3Mut: E184A/D185A/E190A; API5 4Mut: D145A/E184A/D185A/E190A

FGF2 Mut: C211S/C229S/R262A/T263A/K271A (Expressed in *E. coli*); FGF2 3Mut: R262A/T263A/K271A (Expressed in mammalian cells)

Table S2. Minimum Information for Publication of Quantitative Real time PCR Experiments (MIQE) for gene expression analyses

ITEM TO CHECK	IMPORTANCE	CHECKLIST
**Experimental design**		
Definition of experimental and control groups	E	RNAs were extracted from HeLa cells. RT-qPCRs were carried out to measure RNAs of described target genes in each sample.
Number within each group	E	3
Assay carried out by the core or investigator's laboratory?	D	investigator's laboratory
Acknowledgment of authors' contributions	D	
**Sample**		
Description	E	Total/nuclear/cytosolic RNAs isolated from HeLa cells
Volume/mass of sample processed	D	Total RNA extraction: 2.0 x 10^6 cells / sample<br>Cytosol / nuclear RNA fractionation: 2.0 x 10^6 cells / sample
Microdissection or macrodissection	E	Not applicable
Processing procedure	E	Not applicable
If frozen, how and how quickly?	E	Not applicable
If fixed, with what and how quickly?	E	Not applicable
Sample storage conditions and duration (especially for FFPE[2] samples)	E	When RNAs were extracted, RT-qPCRs were carried out immediately.
**Nucleic acid extraction**		
Procedure and/or instrumentation	E	Total, nuclear, and cytoplasmic RNAs were extracted using the RNA subcellular Isolation Kit (Active Motif) according to manufacturer's protocol. A treatment of DNase I was added before washing step.
Name of kit and details of any modifications	E	RNA Subcellular Isolation Kit (Active Motif)<br>RNase-Free DNase Set (Qiagen)
Source of additional reagents used	D	
Details of DNase or RNase treatment	E	RNase-Free DNase Set (Qiagen) was treated in each RNA column for RT 15 minutes before washing step.
Contamination assessment (DNA or RNA)	E	No amplification was detected in no RT samples through PCR and agarose gel-electrophoresis.
Nucleic acid quantification	E	Agilent RNA 6000 Nano Kit / Agilent 2100 bioanalyzer (Agilent Technologies)
Instrument and method	E	Agilent RNA 6000 Nano Kit / Agilent 2100 bioanalyzer (Agilent Technologies)
Purity (A_{260}/A_{280})	D	
Yield	D	
RNA integrity: method/instrument	E	Agilent RNA 6000 Nano Kit / Agilent 2100 bioanalyzer (Agilent Technologies)
RIN/RQI or C_q of 3′ and 5′ transcripts	E	≥ 8.0
Electrophoresis traces	D	
Inhibition testing (C_q dilutions, spike, or other)	E	Not examined
**Reverse transcription**		
Complete reaction conditions	E	The cDNA was synthesized using AMV Reverse Transcriptase XL (Takara Bio). Briefly, each RNAs were mixed with 8μl dNTP Mix (10mM), 2μl of random hexamers (100μM), 2μl of RT buffer (10X), 0.5μl of RTase, and DEPC-treated water up to 20μl. Reactions were incubated in a Mastercycler nexus gradient (Eppendorf, Germany) at 42 °C for 60 min, 96 °C for 5 min to stop the reaction.
Amount of RNA and reaction volume	E	Average of total RNA concentration: ≈700ng / μl; template volume: 1μl; total cDNA reaction volume: 20μl.<br>Because it is an experiment to observe the quantitative ratio of RNA present in the nucleus and cytoplasm, the volume was constant rather than the total amount of RNA.
Priming oligonucleotide (if using GSP) and concentration	E	Random hexamer primers (final concentration 10μM)

Reverse transcriptase and concentration	E	AMV Reverse Transcriptase XL (Takara Bio), 0.25 U / µl
Temperature and time	E	Specified in “Complete reaction conditions”
Manufacturer of reagents and catalogue numbers	D	
C_qs with and without reverse transcription	D	
Storage conditions of cDNA	D	
**qPCR target information**		
If multiplex, efficiency and LOD of each assay	E	Not applicable
Sequence accession number	E	MYC (NM_002467.6), CCND1 (NM_053056.3), MALAT1 (NR_002819.4)
Location of amplicon	D	
Amplicon length	E	MYC (286 bp), CCND1 (159 bp), MALAT1 (620 bp)
In silico specificity screen (BLAST, and so on)	E	Not examined
Pseudogenes, retropseudogenes, or other homologs?	D	
Sequence alignment	D	
Secondary structure analysis of amplicon	D	
Location of each primer by exon or intron (if applicable)	E	All primers were located in exon of RNA.<br>MYC forward: Exon 2 &3; MYC reverse: Exon 3<br>CCND1 forward: Exon 4 & 5; CCND1 reverse: Exon 5<br>MALAT1: single exon
What splice variants are targeted?	E	c-MYC (transcript variant 1), CCND1 (no variant), MALAT1 (transcript variant 1)
**qPCR oligonucleotides**		
Primer sequences	E	c-MYC forward: 5’ CAGCGACTCTGAGGAGGAAC<br>reverse: 5’ GCTGGTGCATTTTCGGTTGT<br>(286 bp)<br>CCND1 forward: 5’ ATGCCAACCTCCTCAACGAC<br>reverse: 5’ TCTGTTCCTCGCAGACCTCC<br>(159 bp)<br>MALAT1 forward: 5’ GCTCTGTGGTGTGGGATTGA<br>reverse: 5’ CTCGGGCGAGGCGTATTTAT<br>(620 bp)
RTPrimerDB identification number	D	
Probe sequences	D	
Location and identity of any modifications	E	None
Manufacturer of oligonucleotides	D	
Purification method	D	
**qPCR protocol**		
Complete reaction conditions	E	PCR reactions were performed in a Real-time PCR LightCycler 96 (Roche Diagnostics) using a FastStart Essential DNA Green Master Kit (Roche Diagnostics) in final volume of 20 µl. Reaction mix consisted of 1µl cDNA and 2µl of 10uM primer mix (forward and reverse primers).
Reaction volume and amount of cDNA/DNA	E	20µl reaction volume containing 1µl of cDNA reaction mixture.
Primer, (probe), Mg2+, and dNTP concentrations	E	5µM primers; dNTP, Mg2+, and dNTP were included in the FastStart Essential DNA Green Master Kit (Roche Diagnostics)
Polymerase identity and concentration	E	A polymerase was included in the FastStart Essential DNA Green Master Kit (Roche Diagnostics)
Buffer/kit identity and manufacturer	E	FastStart Essential DNA Green Master Kit (Roche Diagnostics)
Exact chemical composition of the buffer	D	
Additives (SYBR Green I, DMSO, and so forth)	E	FastStart Essential DNA Green Master Kit (Roche Diagnostics)
Manufacturer of plates/tubes and catalog number	D	

Complete thermocycling parameters	E	The PCR reactions were initiated with 10 minutes incubation at 95 ℃, followed by 45 cycles of 95 ℃ for 10 seconds, 60 ℃ for 20 seconds and 72 ℃ for 10 seconds.
Reaction setup (manual/robotic)	D	
Manufacturer of qPCR instrument	E	Real-time PCR LightCycler 96 (Roche Diagnostics)
**qPCR validation**		
Evidence of optimization (from gradients)	D	
Specificity (gel, sequence, melt, or digest)	E	Melting curve analysis, ramping from 65℃ to 97℃ with a ramp rate of 0.2℃/cycle.
For SYBR Green I, Cq of the NTC	E	No or negligible Cqs
Calibration curves with slope and y intercept	E	Standard curves were examined by real-time qPCR using serially diluted cDNA as templates.
PCR efficiency calculated from slope	E	PCR efficiency was greater than 95%
CIs for PCR efficiency or SE	D	
r2 of calibration curve	E	R2 value was greater than 0.97
Linear dynamic range	E	Sample detection was within the values in the standard curve.
Cq variation at LOD	E	All assays were performed within the linear range of the standard curve.
CIs throughout range	D	
Evidence for LOD	E	All assays were performed within the linear range of the standard curve.
If multiplex, efficiency and LOD of each assay	E	Not applicable
**Data analysis**		
qPCR analysis program (source, version)	E	LightCycler 96 SW 1.1, Excel 2013
Method of Cq determination	E	LightCycler 96 SW 1.1
Outlier identification and disposition	E	Not applicable
Results for NTCs	E	At or below detection limit in at least 40 cycles
Justification of number and choice of reference genes	E	MALAT1 was used as a nuclear marker.
Description of normalization method	E	% of Input = 100 x [2 ^ (Ct total RNA – Ct RNA fraction)]
Number and concordance of biological replicates	D	
Number and stage (reverse transcription or qPCR) of technical replicates	E	3
Repeatability (intraassay variation)	E	LightCycler 96 SW 1.1, Excel 2013
Reproducibility (interassay variation, CV)	D	
Power analysis	D	
Statistical methods for results significance	E	Biological replicates
Software (source, version)	E	Excel 2013
Cq or raw data submission with RDML	D	

Table S3. Antibodies used in this study

Antibody	Manufacturer	Catalog no.
Anti-API5	Abcam	Ab65836
Anti-FGF2	Santa Cruz Biotechnology	sc-74412
Anti-THOC2	Abcam	Ab129485
Anti-UAP56 (DDX39B)	Invitrogen	PA5-27427
Anti-SARNP	Sigma-Aldrich	HPA030902
Anti-eIF4E	Cell Signaling Technology	9742
Anti-LRPPRC	Abcam	Ab97505
Anti-Cyclin D1	Santa Cruz Biotechnology<br>Abcam	sc-246<br>ab134175
Anti-c-MYC	Cell Signaling Technology	9402
Anti-HA	Santa Cruz Biotechnology/Cell Signaling Technology	Sc-7392/3724
Anti-FLAG	Cell Signaling/Sigma-Aldrich	2368/F1804
Anti-β-ACTIN	Sigma-Aldrich	A2228
Anti-His tag	Applied Biological Materials (abm)	G020
Anti-GST tag	Applied Biological Materials (abm)	G018
Goat anti-mouse IgG, HRP-conjugated	Pierce	#31430
Goat anti-rabbit IgG, HRP-conjugated	Pierce	#31460
Alexa Fluor 594-conjugated secondary Ab	Thermo Fisher	mouse A11032, rabbit A11037
Alexa Fluor 568-conjugated secondary Ab	Thermo Fisher	mouse A11004, rabbit A11011
Alexa Fluor 488-conjugated secondary Ab	Thermo Fisher	mouse A11001, rabbit A11008

Table S4. Interface residues of FGF2 involved in API5 or heparin interaction[a]

FGF2–API5 (PDB entry: 6L4O)[b,c]					FGF2–heparin (PDB entry: 1FQ9)			
Residue no. based on HMW FGF2[d]	Residue no. based on LMW FGF2[e]	Residue no. in other PDB[f]	Interaction type[g]	Interacting residues in API5	Residue no. based on HMW FGF2	Residue no. based on LMW FGF2	Residue no. in PDB file	Interaction type
Asn169	Asn36	Asn27	H	Asp185	Asn169	Asn36	Asn27	H
Gly170	Gly37	Gly28			Gly170	Gly37	Gly28	H
Arg181	Arg48	Arg39			Lys261	Lys128	Lys119	S
Arg223	Arg90	Arg81	HS	Asp222	Arg262	Arg129	Arg120	HS
Lys261	Lys128	Lys119			Thr263	Thr130	Thr121	H
Arg262	Arg129	Arg120	HS	Asp145, Asp185, Glu190	Lys267	Lys134	Lys125	S
Thr263	Thr130	Thr121	H	Arg237	Lys271	Lys138	Lys129	S
Gln265	Gln132	Gln123			Gln276	Gln143	Gln134	H
Tyr266	Tyr133	Tyr124			Lys277	Lys144	Lys135	HS
Lys267	Lys134	Lys125	HS	Asp185	Ala278	Ala145	Ala136	H
Leu268	Leu135	Leu126						
Lys271	Lys138	Lys129	HS	Glu184, Glu219				
Lys277	Lys144	Lys135	HS	Asp145				
Ala278	Ala145	Ala136						

[a]Analyzed using the PISA (http://www.ebi.ac.uk/msdsrv/prot_int/cgi-bin/piserver) or FLIP server (https://projects.biotec.tu-dresden.de/plip-web) for protein-protein or protein-ligand interactions, respectively(6,7).

[b]All residues of API5 participating in the API5–FGF2 interaction: Gly143, Glu144, Asp145, Arg148, Leu 183, Glu184, Asp185, Val186, Thr187, Gly188, Glu190, Glu219, Gln220, Asp222, Glu224, Gln225, Asn228, Ser230, Asp231, and Arg237 (20 residues).

[c]All residues of FGF2 participating in the API5–FGF2 interaction: Asn169, Gly170, Arg181, Arg223, Lys261, Arg262, Thr263, Gln265, Tyr266, Lys267, Leu268, Lys271, Lys277, and Ala278 (14 residues).

[d]Residue numbers are based on the longest amino acid sequence (UniProtKB—P09038-4, 288 aa).

[e]Residue numbers are based on the shortest LMW FGF2 amino acid sequence (UniProtKB—P09038-2, 155 aa).

[f]PDB entry 1FQ9; FGF2–FGFR1–heparin complex structure.

[g]H, hydrogen bond; S, salt bridge; blank, interface residue with no other distinguishing characteristic.

Table S5. Residues of FGF2 involved in the FGFR1 interaction (analyzed with PDB entry 1FQ9)[a]

Residue no. based on HMW FGF2[b]	Residue no. based on LMW FGF2[c]	Residue no. in PDB file[d]	Interaction type[e]	Residue no. based on HMW FGF2	Residue no. based on LMW FGF2	Residue no. in PDB file	Interaction type
His158	His25	His16	S	Arg202	Arg69	Arg60	H
Phe159	Phe26	Phe17	H	Gly203	Gly70	Gly61	
Lys160	Lys27	Lys18		Val205	Val72	Val63	
Lys163	Lys30	Lys21	H	Ser206	Ser73	Ser64	
Tyr166	Tyr33	Tyr24	H	Tyr215	Tyr82	Tyr73	
Lys168	Lys35	Lys26		Val230	Val97	Val88	
Gly170	Gly37	Gly28		Phe235	Phe102	Phe93	
Gly171	Gly38	Gly29		Glu238	Glu105	Glu96	H
Phe173	Phe40	Phe31		Arg239	Arg106	Arg97	
Arg186	Arg53	Arg44		Leu240	Leu107	Leu98	
Lys188	Lys55	Lys46		Asn243	Asn110	Asn101	
Gln196	Gln63	Gln54		Asn244	Asn111	Asn102	H
Leu197	Leu64	Leu55		Tyr245	Tyr112	Tyr103	
Gln198	Gln65	Gln56	H	Asn246	Asn113	Asn104	H
Ala199	Ala66	Ala57		Leu282	Leu149	Leu140	
Glu200	Glu67	Glu58	H	Pro283	Pro150	Pro141	
Glu201	Glu68	Glu59		Met284	Met151	Met142	

[a]Analyzed using the PISA (http://www.ebi.ac.uk/msdsrv/prot_int/cgi-bin/piserver) for protein-protein interaction.

[b]Residue numbers are based on the longest HMW FGF2 amino acid sequence (UniProtKB—P09038-4, 288 aa).

[c]Residue numbers are based on the shortest LMW FGF2 amino acid sequence (UniProtKB—P09038-2, 155 aa).

[d]PDB entry 1FQ9; FGF2–FGFR1–heparin complex structure.

[e]H, hydrogen bond; S, salt bridge; blank, interface residue with no other distinguishing feature.

Table S6. Summary of binding affinity and kinetic parameter measurements of protein-protein interaction by SPR[a]

Interaction	K_d	k_{on} ($M^{-1}s^{-1}$)	k_{off} (s^{-1})	$t_{1/2}$ (s)
API5 WT–FGF2[b]	676(±5) nM	7.01(±0.05)×10^3	4.74(±0.03)×10^{-3}	146
API5 WT–FGF2 Mut[b]	1.51(±0.01) μM	1.66(±0.01)×10^3	2.51(±0.02)×10^{-3}	276
API5 3Mut[c]–FGF2	4.20(±0.10) μM	2.26(±0.06)×10^3	9.40(±0.10)×10^{-3}	74
API5 3Mut–FGF2 Mut	17.3(±0.4) μM	1.90(±0.04)×10^2	3.28(±0.03)×10^{-3}	211
UAP56–API5	1.37(±0.00) μM	3.05(±0.00)×10^3	4.19(±0.03)×10^{-3}	165
UAP56–FGF2	382(±6) nM	1.22(±0.02)×10^4	4.65(±0.05)×10^{-3}	149
UAP56–API5+FGF2	199(±3) nM	1.42(±0.02)×10^4	2.83(±0.02)×10^{-3}	245

[a]Results for Figure 1F and Figure 4E.

[b]FGF2 is C211S/C229S and FGF2 Mut is C211S/C229S/R262A/T263A/K271A where C211S/C229S mutation was introduced for the expression of recombinant FGF2 in *E. coli*, and R262A/T263A/K271A mutation contributes in the API5 interaction.

[c]API5 3Mut is a E184A/D185A/E190A mutant.

Table S7. Full list of API5 interaction partners by immunoprecipitation and proteomics analysis

Protein name
BCLAF1, TPM1, TPM2, TPM3, DDX39A, MPRIP, SLC25A6, JUP, SARNP, CTNNA1, PALLD, GNAS, RTN4, VARS, LRPPRC, PFKP, TNPO1, YWHAH, PRKACA, AP3B1, GID8, HLA-A, PSMC2, DYNLL1, HSPH1, RALA, CSE1L, TRIP13, TPR, UQCRC2, FOXRED1, SEC22B, TXNDC5, TMED10, CRNKL1, DDX46, AP2B1, WDR26, CYB5R3, PPIL1, TJP1, EPB41L2, ANP32A, SLC1A5, SORBS2, KRAS, CD44, RAD21, HDAC1, ZFR, PSMD4, ARCN1, PSMA2, GART, RAB7A, SMNDC1, ARPC5L, RNF219, TMSB4X, TECR, AIDA, ALDH1B1, BAG3, KANK2, DDX1, SEPT11, PSMC4, MRPS27, CHCHD2P9, SLC25A13, PDE3A, LYAR, NCAPH, DNAJB11, EIF5, NARS, ARPC5, UQCR10, MGST1, GDI2, CTPS1, SMARCE1, EXOSC2, HIST2H2AB, SSR3, YARS, RBFOX2, LMNB2, OGDH, TMEM33, NOP10, ATP5H, CPSF7, DYNLRB2, CDH2, SEC13, SF3B5, SREK1IP1, PRPF40A, GNA11, MRPL9, ATXN2L, CORO1B, SNRPA, SAFB2, CCDC75(GPATCH1), MRE11A(MRE11), PSMA3, SLIRP, C1QA, PSMB5, LEPRE1(P3H1), PPP2CB, PPP3CB, GRSF1, LSM3, MBNL1, NOL9, MRPL3, MRPS2, CWC22, KIF19, YTHDF3, SPTBN1, POLDIP3, ZC3H11A, ZC3H14, CALU, THOC2, C19orf47, EEF1A2, FYTTD1, C17orf85(NCBP3), THOC1, PLRG1, KRT4, XAB2, KCTD18, SCAF1, DHX8, MFAP1, HLA-B, THOC6, CCDC9, SRRM1, THOC5, CDC40, USP39, RAB14, TWF1, KIF5C, FIP1L1, BUD31, SREK1, TWF2, RANBP10, C15orf52, EIF4E, ALKBH5, FAM107B, POLR2B, RBM27, MARCKSL1, POLR2A, RBM19, SURF6, RANBP9, CCDC12, CPSF1, LSM4, PDCD6IP, CAAP1, RBM25, SMN1(SMN2), BUB3, CPS1, DYNC1I2, ACTL6A, ISY1-RAB43, ICT1(MRPL58), P4HA1, ZC3H13, TNKS1BP1, SYF2, SF1, SEC23B, POLR2H, RAD50, C1orf35, GADD45GIP1, SH3BGRL3, FGF2, UPF3B, POLR2E, RBMX2, KIF13B, PRKRA, LSM6, YTHDC1, GCFC1(PAXBP1), PURA, MRPS16, FAM207A, THOC3, STK24, AKAP17A, TROVE2, MBD2, NEXN, NDUFB10, HDX, PCNP, CUL2, DLAT, TCOF1, KPNA3, HIVEP3, TACC3, SLC4A1AP, FOXG1, CSTB, SAP18, TFIP11, COTL1, GPR56(ADGRG1), TK1, SYNRG, ATP5D, OSMR, DAGLA, RAE1, NLRP9, IL18, UTRN, CYP4F2

Table S8. Selected functional classification of API5 interaction partners by proteomics analysis[a]

Categories	Functional Annotation	p-Value	Molecules	No. of Molecules
Molecular Transport, RNA Trafficking	Nuclear export of RNA	1.13E-13	ALKBH5, DDX39A, EIF4E, FYTTD1, RAE1, SARNP, THOC1, THOC2, THOC3, THOC5, THOC6, TPR	12
Molecular Transport, RNA Trafficking	Transport of RNA	1.77E-13	ALKBH5, DDX39A, EIF4E, FYTTD1, KIF5C, RAE1, SARNP, THOC1, THOC2, THOC3, THOC5, THOC6, TPR	13
Molecular Transport, RNA Trafficking	Transport of mRNA	5.06E-13	ALKBH5, DDX39A, EIF4E, FYTTD1, KIF5C, RAE1, SARNP, THOC1, THOC2, THOC3, THOC5, THOC6	12
Molecular Transport, RNA Trafficking	Nuclear export of mRNA	5.41E-13	ALKBH5, DDX39A, EIF4E, FYTTD1, RAE1, SARNP, THOC1, THOC2, THOC3, THOC5, THOC6	11
RNA Post-Transcriptional Modification	Processing of RNA	8.31E-13	AKAP17A, ALKBH5, CDC40, CPSF1, CRNKL1, DDX39A, DHX8, GRSF1, LSM3, LSM4, LSM6, MBNL1, PLRG1, POLR2A, PRKACA, RBFOX2, RBM25, SAP18, SNRPA, SREK1, SRRM1, THOC1, USP39, YTHDC1	24
RNA Post-Transcriptional Modification	Processing of mRNA	7.39E-11	ALKBH5, CDC40, CPSF1, CRNKL1, DDX39A, GRSF1, LSM3, MBNL1, PLRG1, POLR2A, PRKACA, RBFOX2, RBM25, SAP18, SNRPA, USP39, YTHDC1	17
RNA Post-Transcriptional Modification	Splicing of RNA	3.68E-10	AKAP17A, CDC40, CRNKL1, DDX39A, DHX8, LSM4, LSM6, MBNL1, PLRG1, RBFOX2, RBM25, SAP18, SREK1, SRRM1, USP39, YTHDC1	16

[a]The program IPA (Qiagen Bioinformatics) was used for analysis.

Supplementary References

1. Kim, H.Y., Kim, D.K., Bae, S.H., Gwak, H., Jeon, J.H., Kim, J.K., Lee, B.I., You, H.J., Shin, D.H., Kim, Y.H. *et al.* (2018) Farnesyl diphosphate synthase is important for the maintenance of glioblastoma stemness. *Exp Mol Med*, **50**, 1-12.
2. Kramer, A., Green, J., Pollard, J., Jr. and Tugendreich, S. (2014) Causal analysis approaches in Ingenuity Pathway Analysis. *Bioinformatics*, **30**, 523-530.
3. Song, B., Kim, D.K., Shin, J., Bae, S.H., Kim, H.Y., Won, B., Kim, J.K., Youn, H.D., Kim, S.T., Kang, S.W. *et al.* (2018) OCT4 directly regulates stemness and extracellular matrix-related genes in human germ cell tumours. *Biochem Biophys Res Commun*, **503**, 1980-1986.
4. Robert, X. and Gouet, P. (2014) Deciphering key features in protein structures with the new ENDscript server. *Nucleic Acids Res*, **42**, W320-324.
5. Larkin, M.A., Blackshields, G., Brown, N.P., Chenna, R., McGettigan, P.A., McWilliam, H., Valentin, F., Wallace, I.M., Wilm, A., Lopez, R. *et al.* (2007) Clustal W and Clustal X version 2.0. *Bioinformatics*, **23**, 2947-2948.
6. Krissinel, E. and Henrick, K. (2007) Inference of macromolecular assemblies from crystalline state. *J Mol Biol*, **372**, 774-797.
7. Salentin, S., Schreiber, S., Haupt, V.J., Adasme, M.F. and Schroeder, M. (2015) PLIP: fully automated protein-ligand interaction profiler. *Nucleic Acids Res*, **43**, W443-447.
